# Identification of a pharyngeal mucosal lymphoid organ in zebrafish and other teleosts: Tonsils in fish?

**DOI:** 10.1126/sciadv.adj0101

**Published:** 2023-11-01

**Authors:** Julien Resseguier, Mai Nguyen-Chi, Jens Wohlmann, Dimitri Rigaudeau, Irene Salinas, Stefan H. Oehlers, Geert F. Wiegertjes, Finn-Eirik Johansen, Shuo-Wang Qiao, Erling O. Koppang, Bernard Verrier, Pierre Boudinot, Gareth Griffiths

**Affiliations:** ^1^ Section for Physiology and Cell Biology, Departments of Biosciences and Immunology, University of Oslo, Oslo, Norway.; ^2^ LPHI, CNRS, Université de Montpellier, Montpellier, France.; ^3^ Electron-Microscopy laboratory, Departments of Biosciences, University of Oslo, Oslo, Norway.; ^4^ INRAE, Université Paris-Saclay, IERP, 78350 Jouy-en-Josas, France.; ^5^ Center for Evolutionary and Theoretical Immunology (CETI), Department of Biology, University of New Mexico, Albuquerque, NM, USA.; ^6^ A*STAR Infectious Diseases Labs (A*STAR ID Labs), Agency for Science, Technology and Research (A*STAR), 8A Biomedical Grove, Immunos #05-13, Singapore 138648, Singapore.; ^7^ Aquaculture and Fisheries Group, Department of Animal Sciences, Wageningen University & Research, Wageningen, Netherlands.; ^8^ Section for Physiology and Cell Biology, Department of Biosciences, University of Oslo, Oslo, Norway.; ^9^ Department of Immunology, Institute of Clinical Medicine, University of Oslo, Oslo, Norway.; ^10^ Unit of Anatomy, Faculty of Veterinary Medicine, Norwegian University of Life Sciences, Ås, Norway.; ^11^ Laboratory of Tissue Biology and Therapeutic Engineering, UMR 5305, IBCP, CNRS, University Lyon 1, Lyon, France.; ^12^ Université Paris-Saclay, INRAE, UVSQ, Virologie et Immunologie Moléculaires, Jouy-en-Josas, France.

## Abstract

The constant exposure of the fish branchial cavity to aquatic pathogens causes local mucosal immune responses to be extremely important for their survival. Here, we used a marker for T lymphocytes/natural killer (NK) cells (ZAP70) and advanced imaging techniques to investigate the lymphoid architecture of the zebrafish branchial cavity. We identified a sub-pharyngeal lymphoid organ, which we tentatively named “Nemausean lymphoid organ” (NELO). NELO is enriched in T/NK cells, plasma/B cells, and antigen-presenting cells embedded in a network of reticulated epithelial cells. The presence of activated T cells and lymphocyte proliferation, but not V(D)J recombination or hematopoiesis, suggests that NELO is a secondary lymphoid organ. In response to infection, NELO displays structural changes including the formation of T/NK cell clusters. NELO and gill lymphoid tissues form a cohesive unit within a large mucosal lymphoid network. Collectively, we reveal an unreported mucosal lymphoid organ reminiscent of mammalian tonsils that evolved in multiple teleost fish families.

## Teaser

An unknown prominent mucosal lymphoid organ has been identified in the branchial cavity of zebrafish, and other teleost fish.

## INTRODUCTION

The survival of pluricellular organisms requires defense mechanisms against infections at barrier tissues, the interface between the environment and the host. The emergence of adaptive immunity, approximately 500 million years ago, marked an important milestone in the defense against pathogens. Adaptive immunity is based on the clonal selection of lymphocytes expressing somatically diversified genes encoding antigen (Ag) receptors. The production and differentiation of naïve B or T cells occur in primary lymphoid organs, naïve lymphocytes then relocate to secondary lymphoid organs where adaptive immune responses are initiated. Both primary and secondary lymphoid organs are constitutive and develop at predetermined locations (*1*). During evolution, the emergence of secondary lymphoid organs has been essential in facilitating adaptive immune responses by providing an organizational framework favoring the colocalization of Ags and Ag-specific lymphocytes required for the efficient induction of antibody-mediated responses (*2*). The evolution of lymphoid structures and adaptive immunity among vertebrates have been extensively studied (*3*–*10*). Unexpectedly, despite the fact that teleost fish represent approximately half of all vertebrate species (*11*, *12*), their immune system has received little attention, which is particularly noteworthy given the importance of these fish as a food source for humans and animals (*13*).

The basic components of the immune system of teleost fish share many similarities with mammals (*14*, *15*). Most of the cells of the mammalian innate and adaptive immune systems have also been identified in teleost fish, including granulocytes (*16*), innate lymphoid cells (*17*), T cells (*18*), B cells (*19*), and Ag-presenting cells such as macrophages (*20*). Key molecular mechanisms involved in the detection of pathogens (*21*) and in the regulation of immune responses (*22*) are also shared across jawed vertebrates. Teleost fish have two known primary lymphoid organs: (i) the thymus, where T cells develop and mature (*23*), and (ii) the kidney, a site where hematopoiesis occurs and where B cell precursors develop. The anterior part of the teleost fish kidney, the pronephros (also named “head-kidney”), is also a prominent site of immune activity associated with secondary lymphoid organ functions (*24*). However, in fish, it is the spleen that is considered the main systemic secondary lymphoid organ (*25*). No lymph nodes nor tonsil equivalents have been observed in teleost fish. Although no clear counterparts of mammalian germinal centers have been identified in teleost fish, the stimulation of the fish immune system can nevertheless induce the development of structures such as melano-macrophage centers (*26*).

In both fish and mammals, mucosal tissues provide an extensive surface that connects the organism with the outside world, thereby facilitating critical functions such as nutrient absorption and gas exchange, but at the same time increasing exposure to pathogens. As in mammals, fish mucosae are protected by multiple “mucosa-associated lymphoid tissues” (MALTs) which function in the immune surveillance of the mucosal interface (*27*). The main fish MALTs are located in the gut (GALT), the skin (SALT), the nostril (NALT), and the gills (GIALT) (*28*–*31*). Recent studies have also reported the existence of a MALT associated with the mouth and the pharynx (*32*, *33*). In mammals, the organization of MALTs is well defined into regions where disorganized immune cells are scattered, hence forming a diffuse mucosal immune system, and into organized lymphoid aggregates such as Peyer’s patches in the gut and Waldeyer’s ring of tonsils in the nasopharyngeal area (*34*). In contrast, the fish mucosal immune system has long been perceived as a set of scattered immune cells spread along mucosal territories (*19*, *27*). However, such a mucosal immune system may be difficult to reconcile with the evolutionary pressures exerted by the high concentration of microbes in aquatic environments (*35*). The absence of organized mucosal lymphoid structures in teleost fish has been challenged by recent discoveries, such as the identification of the interbranchial lymphoid tissue (ILT) within the gills of Atlantic salmon (*Salmo salar*) in 2008 (*36*). This result illustrated the need for further investigation of teleost lymphoid organization. Toward this goal, we took advantage of the zebrafish as a well-established model system (*37*). While the zebrafish has one of the best-characterized immune systems among teleost fish, it is also ideally suited for imaging, for whole-organism investigations, and benefits greatly from numerous molecular tools, including many publicly available genetically modified strains.

In a previous study (*31*), using high-resolution three-dimensional (3D) imaging of zebrafish gills, we characterized the organization of the GIALT and identified its compartmentalization into segments where immune cells are scattered and two lymphoid aggregates that display features of secondary lymphoid organs: the ILT and a recently identified lymphoid tissue that we called the amphibranchial lymphoid tissue (ALT). Our findings revealed a higher degree of organization of fish MALTs and support our contention that there is still much to learn about the organization of the fish immune system. This certainly applies to the branchial cavity (also named gill chambers or pharyngeal cavities), the anatomy of which remains poorly understood. The branchial cavity consists of two chambers, one on each side of the head, that are bridged by the pharynx in the middle and are open to the outside via the operculum slits. The region below the pharynx that separates the gill chambers is called the sub-pharyngeal isthmus. The whole branchial cavity is lined by a nonkeratinized squamous “pharyngeal” epithelium (*7*, *38*), which will be named “cavo-branchial epithelium” in the present study to distinguish it from the histologically distinct epithelium that covers the pharynx. Each zebrafish gill chamber displays a set of four gill arches that each comprises two ALTs and one ILT (*31*). Last, a thymus lobe is located on the roof of each gill chamber. The overall anatomy of the branchial cavity and the gills are illustrated in figs. S1 and S2.

To investigate the whole branchial cavity, we used cryosections of adult zebrafish heads in which we labeled tissue structures with fluorescent probes and then identified lymphoid structures using an antibody targeting a highly conserved epitope of the kinase ZAP70, a marker of T/NK cells. Our observations revealed a prominent lymphoid organ along the sub-pharyngeal region of the branchial cavity that, to the best of our knowledge, had not been previously described, and which we named the “Nemausean lymphoid organ” (NELO).

## RESULTS

### Identification of the Nemausean lymphoid organ, a previously undescribed lymphoid structure inside the branchial cavity

As the zebrafish branchial cavity constitutes a complex anatomical territory, we conducted high-resolution 3D multi-field-of-view imaging of whole branchial cavity cryosections (30 μm) from adults. These sections were stained with fluorescent phalloidin to label F-actin and 4′,6-diamidino-2-phenylindole (DAPI) to stain DNA, facilitating the identification of tissue structures. We focused on the immunolabeling of “Zeta-chain–associated protein kinase 70” (ZAP70), a T cell/natural killer (NK) cell marker (*39*), to reveal the organization of lymphoid tissues. The anti-ZAP70 monoclonal antibody (99F2) has previously been validated in zebrafish (*40*) and displays a good affinity for its epitope across many species (*31*, *41*). Additional data on the specificity of the anti-ZAP70 antibody are presented in fig. S3.

**Fig. 1.**
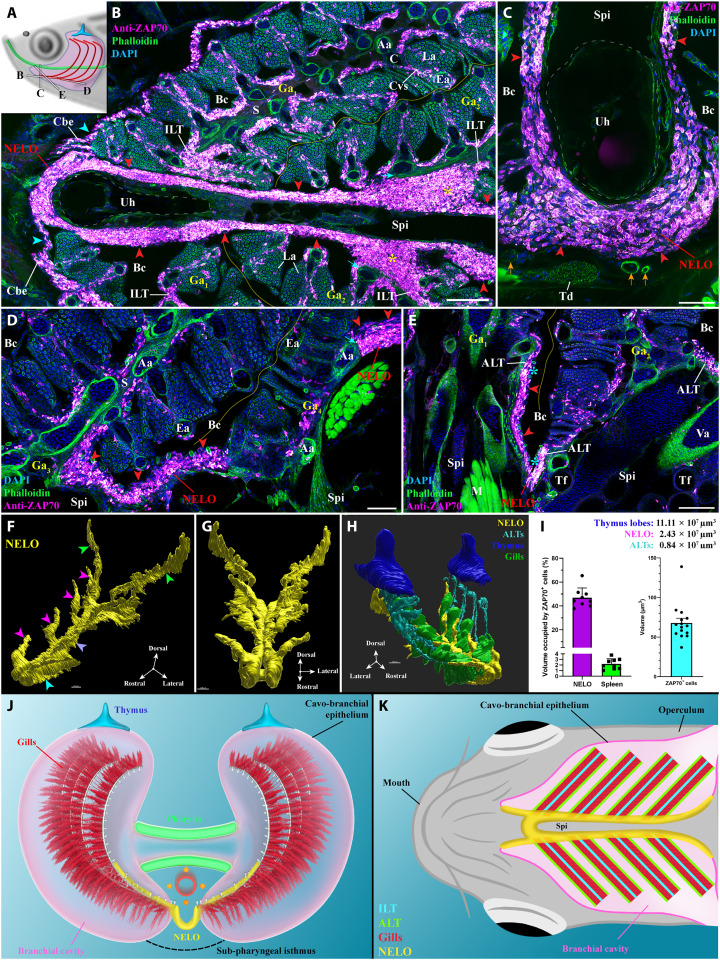
General organization and localization of NELO in zebrafish. (**A**) Scheme illustrating the different orientations of the NELO images acquired from 30-μm whole adult zebrafish head cryosections, and the position of the thymus (blue), pharynx (green), and gills (red). (**B** to **E**) NELO (red arrowheads) wraps around the urohyal bone (B and C) and extends along the sub-pharyngeal isthmus toward the posterior end of the gill chambers (D). NELO is connected to the ILTs [(B), yellow stars], the ALTs [(E), cyan stars], and the cavo-branchial epithelium [(A), cyan arrowheads]. NELO is close to the gills afferent arteries [(B), cyan arrows] and other endothelial vessels [(C), orange arrows]. (**F** and **G**) NELO 3D reconstruction obtained by serial confocal tomography of a ZAP70-labeled wholemount head of zebrafish (15 wpf) and its segmentation into four anatomic regions: the anterior area wrapped around the urohyal bone (cyan arrowhead), antler-like protrusions (magenta arrowheads), the core (blue arrow), and the posterior end (green arrowheads). (**H**) Three-dimensional reconstruction of NELO (yellow), ALTs (cyan), thymus lobes (blue), and the ventral extremity of gill arches (green). (**I**) Volumes of the 3D reconstructed lymphoid structures, a fraction of the volume occupied by T/NK cells in NELO and the spleen, and the average volume of a single T/NK cell. (**J** and **K**) Illustrations of NELO’s localization as observed from the front (J) or from below (K). Illustrations made by Ella Maru studio and K. Zulkefli. Annotations: Aa, afferent artery; ALT, amphibranchial lymphoid tissue; Bc, branchial cavity; C, cartilage; Cbe, cavo-branchial epithelium; Cvs, central venous sinus; Ea, efferent artery; Ga, gill arch; ILT, interbranchial lymphoid tissue; La, lamellae; M, muscles; NELO, Nemausean lymphoid organ; S, septum; Spi, sub-pharyngeal isthmus; Td, tendon; Tf, thyroid follicle; Uh, urohyal bone; Va, ventral aorta. Scale bars, 150 (H), 100 (B, and E to G), 50 (D), and 40 μm (C).

When exploring the lower region of the adult zebrafish branchial cavity in cryosections at various orientations (Fish, *N* = 10) (Fig. 1A), we found a previously not identified mucosal lymphoid organ below the pharynx, at the convergence of the gill arches with the sub-pharyngeal isthmus. We tentatively named it the Nemausean lymphoid organ (NELO) inspired by the Gallic-Roman “Nemausus - Nemausicae” mythology associated with protection, water, and healing. NELO was present in all analyzed zebrafish.

Analysis of the cryosections revealed that NELO constitutes a large structure enriched in ZAP70-positive cells located within the squamous mucosal epithelium lining the sub-pharyngeal isthmus, a region under the pharynx that separates the two gill chambers (Fig. 1, B to E, red arrowheads). NELO wraps around the urohyal bone at the anterior end of the branchial cavity (Fig. 1, B and C) before extending along each side of the sub-pharyngeal isthmus until it reaches the posterior end of the branchial cavity (Fig. 1D). Along its length, NELO is connected to all 24 gill lymphoid aggregates, i.e., the 8 ILTs (Fig. 1B, yellow stars) and the 16 ALTs (Fig. 1E, cyan stars). We could not define any clear separation between ILT/ALT and NELO at these connection sites, suggesting that lymphoid structures of the branchial cavity may function as a single integrated unit. Other images displaying NELO and its direct connection with gill lymphoid aggregates are shown in fig. S4 (A to D). In addition, the existence of NELO could be confirmed based on the expression of the kinase *lck* gene using the *Tg(lck:EGFP)* transgenic adult zebrafish (*18*, *42*), in which enhanced green fluorescent protein (EGFP) is highly expressed in T lymphocytes (fig. S4, E and F).

### 3D structure of NELO

Our data indicated that NELO exhibits a sophisticated architecture that is difficult to fully capture from cryosections. We therefore produced a reconstruction of NELO’s structure in three dimensions using serial confocal tomography from whole young adult wild-type zebrafish heads [15 weeks postfertilization (wpf)]. Combining successive vibratome sections and confocal microscopy, this approach enabled the assembly of a NELO 3D structure from over 700 imaged layers, thereby defining NELO boundaries using the distribution of the ZAP70 signal (Fig. 1, F and G). This accurate representation revealed the segmentation of NELO into four distinct anatomical subregions: (i) the anterior-most region that wraps around the urohyal bone (Fig. 1F, cyan arrowhead), (ii) the four “antler-like” protrusions that each connect with two ALTs (Fig. 1F, magenta arrowheads), (iii) the core that extends along the sub-pharyngeal isthmus (Fig. 1F, blue arrowhead), and (iv) the posterior end that starts after the fourth set of gill arches and extends toward the operculum opening (Fig. 1F, green arrowheads). The 3D reconstruction of the thymus lobes (blue), the 16 ALTs (cyan), and the ventral extremity of the eight gill arches (green) allowed us to interpret NELO (yellow/magenta) in the spatial context of the fish, and in particular of the branchial cavity (Fig. 1H and movies S1 to S4). This representation illustrated the localization of NELO along the ventral axis of the fish head and the continuity between NELO antler-like protrusions with all ALTs. The localization of NELO within the branchial cavity is further shown Fig. 1 (J and K).

We then estimated the volume of NELO based on the 3D reconstruction shown in Fig. 1 (F to H). In this 15 wpf zebrafish, NELO had a volume of 2.4 × 10^7^ μm^3^, which is smaller than the thymus (11.1 × 10^7^, 5.5 × 10^7^, and 5.6 × 10^7^ μm^3^ for each lobe); at this stage, the thymus has just started to involute. Using a stereology approach and 3D reconstruction, we estimated that T/NK cells occupy 46.8% of NELO’s volume (sections: *N* = 9 obtained from three fishes) for an average volume of 67.7 μm^3^ per ZAP70-positive cell (3D reconstructed cells: *N* = 15 obtained from three fishes) (Fig. 1I). In this 15 wpf zebrafish, NELO would then contain around 165,000 T/NK cells. In comparison, the number of spleen T/NK cells, assessed using a similar approach (sections: *N* = 9 obtained from three fishes) and a rough estimation of the spleen volume, was around 120,000 T/NK cells (2.2% of the spleen’s volume). Noteworthy, the overall volume of the 16 ALTs was estimated to be 0.84 × 10^7^ μm^3^, therefore indicating that the ALTs represent much smaller structures compared to NELO. Collectively, these data indicate that NELO is a prominent structure of the branchial cavity and provide a first line of evidence that it constitutes a separate organ.

### NELO: A mucosal lymphoid organ

**Fig. 2.**
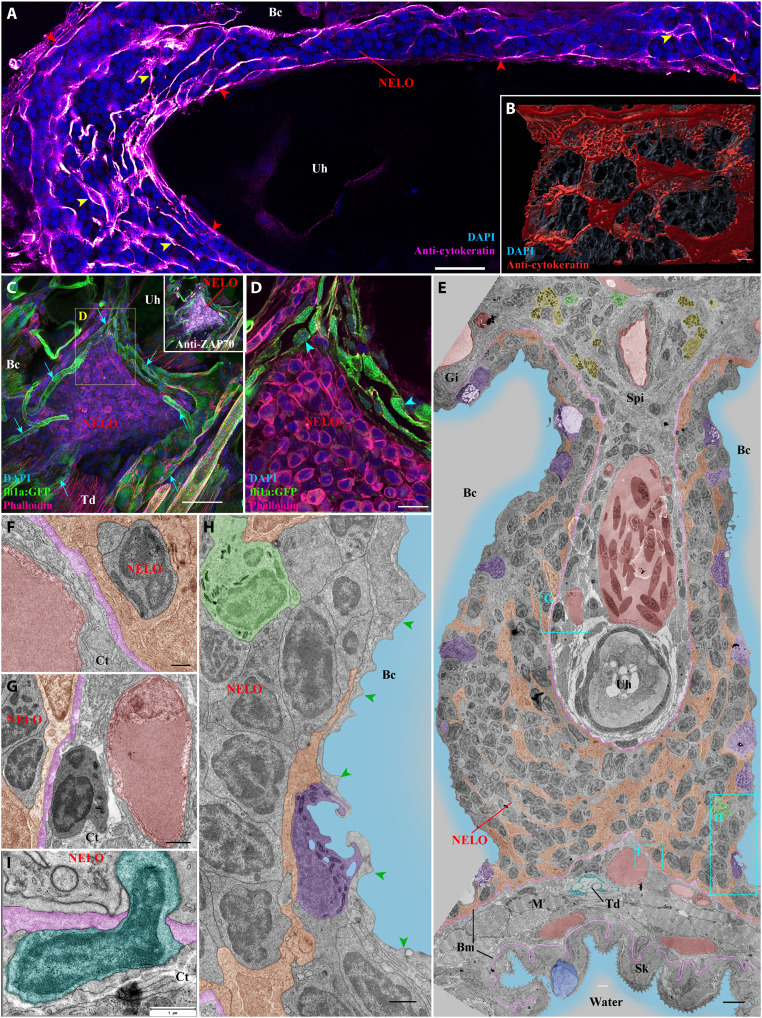
Detailed structural organization of the adult zebrafish NELO. (**A**) Cryosection labeled with anti-cytokeratin antibodies (magenta hot) revealing a network of reticulated epithelial cells within (yellow arrowheads) and bordering NELO (red arrowheads). (**B**) Three-dimensional reconstruction illustrating the network of reticulated epithelial cells in red. (**C**) Three-dimensional imaging of a cryosection displaying NELO from a fli:GFP zebrafish, in which endothelial vessels are fluorescent (green). Numerous vessels are wrapped around NELO (cyan arrows). (**D**) Optical section from (C) highlighting cuboidal-shaped endothelial cells (cyan arrowheads). (**E** to **H**) Ultrastructure map of a 9 wpf zebrafish NELO transversally sectioned at the urohyal bone acquired by transmission electron microscopy. Several structures have been highlighted: reticulated epithelial cells (orange), mucous cells (dark blue), water (light blue), ionocytes (purple), endothelial vessels (burgundy red), basement membrane (pink), neutrophils (green), basophils/mast cells (yellow), tenocytes (dark blue-green), and pavement cells (green arrowheads). (F) to (H) represent zoomed areas from (E). (**I**) Cell (dark blue-green) observed across the basement membrane (pink) separating NELO from the surrounding connective tissue. Annotations: Bm, basement membrane; Gi, Gills; Sk, Skin. Scale bars, 30 μm (C), 20 μm (A), 10 μm (D), 4 μm (E), 3 μm (B), 1 μm (G, H, and I), and 500 nm (F).

We then asked whether the structural organization of NELO at the cellular level was consistent with the known organization of lymphoid organs and tissues. A hallmark of structured lymphoid aggregates, such as the thymus, lymph nodes, Peyer’s patches, or tonsils in mammals, is the characteristic arrangement of the immune cells within a meshwork of reticulated epithelial cells that acts as an immuno-platform (*43*, *44*). This feature was a key element in classifying teleost fish ILT and ALT as lymphoid tissues (*31*, *36*). We therefore labeled cryosections of NELO with a commonly used cocktail of antibodies to reveal cytokeratins, which are essential constituents of the reticulated epithelial cell cytoskeleton. This immunolabeling revealed a complex network of reticulated epithelial cells at the boundaries (red arrowheads) and within (yellow arrowheads) the anterior segment of NELO (Fig. 2A). Further analysis confirmed that this network extended throughout NELO in its entirety (fig. S5, A and B). Three-dimensional reconstruction of the cytokeratin signal revealed that the arrangement of reticulated epithelial cells constitutes organized pockets of cells that are typical of lymphoid aggregates (Fig. 2B and movie S5). In addition, NELO-reticulated epithelial cells shared low levels of major histocompatibility complex (MHC) class II (*mhc2dab*) (fig. S5, C to F) and hemi-desmosomes (fig. S5G, cyan arrow) with their counterparts from mammalian lymphoid aggregates (*43*, *45*).

To sustain their functions, lymphoid organs require access to oxygen and nutrient supply, as well as mechanisms to facilitate immune cell trafficking. The localization of NELO in close proximity to inhaled water ensures a sustained oxygen supply. The circulatory system functions as a conduit for nutrient delivery and immune cell trafficking throughout the body. The next question was therefore to determine whether NELO is vascularized. For this, we performed 3D imaging of cryosections from an adult zebrafish line in which endothelial cells express a fluorescent protein *Tg(fli1a:EGFP)* (*46*). This experiment revealed numerous endothelial vessels surrounding NELO (Fig. 2C, cyan arrows, and movie S6). However, no endothelial structures were found within the organ itself. Opening up the 3D stacks to look at the optical sections, we found that some of the narrow vessels surrounding NELO were lined by cuboidal endothelial cells (Fig. 2D, cyan arrowheads), which sometimes line fish arteries and heart endocardium (*47*). However, these particular vessels lacked the characteristic layer of smooth muscles that usually surround fish arteries. In humans, cuboidal endothelial cells are a hallmark of the high-endothelial venules that are characteristic of mammalian lymph nodes and tonsils (*48*). Further studies will be necessary to determine whether these endothelial structures constitute blood vessels, conventional lymphatic vessels, or the nonconventional blood/lymphatic “fine” vessels reported in cod almost a century ago by Burne (*49*). In addition to the previously described vessels, NELO also benefits from close proximity to the prominent gill vasculature at its convergence with the gill lymphoid aggregates (Fig. 1, B and D, cyan arrows).

### Ultrastructure of NELO

To continue our investigation of NELO at a higher resolution, we used transmission electron microscopy (TEM) of ultrathin sections and a new data browsing method developed by J. Wohlmann to easily access ultrastructural images at different scales over a large area. The resulting dynamic ultrastructure map allows the user to efficiently navigate within the biological sample. Using this approach, we assembled a detailed map (>1400 micrographs) covering a substantial portion of NELO’s anterior segment in a 9 wpf juvenile zebrafish (Fig. 2E).

The EM data highlighted a number of notable features and provided additional insights into NELO. The network of reticulated epithelial cells was prominent (orange) and the nuclei of these cells were much less electron-dense and more elongated than the nuclei of the neighboring cells (Fig. 2E and fig. S5G). EM analysis confirmed the close proximity of NELO to neighboring endothelial vessels (burgundy red), which were mostly separated by a thin basement membrane (pink) that forms a boundary between NELO and the surrounding connective tissue (Fig. 2, F and G). These observations support our view that NELO constitutes a distinct entity. The ultrastructure map shows unequivocally that NELO was only separated from the outside environment by a single layer of epithelial cells (Fig. 2H). These cells were predominantly pavement cells, which can be identified by their elongated shape and typical actin microridges (green arrowheads). Interspersed between pavement cells were mitochondria-rich cells (ionocytes) (Fig. 2, E and H, purple). This squamous mucosal epithelium was reminiscent of the epithelium that lines the gills (*50*). The EM analysis also revealed the presence of cells that have penetrated the basement membrane bordering NELO (Fig. 2I, cyan), suggesting the existence of cell traffic in or out of NELO.

Whereas the thymus lobes are already present in 3 days postfertilization zebrafish (*18*), we could not detect NELO in 3 wpf zebrafish (fig. S6). Moreover, in the 9 wpf juveniles used for ultrastructure investigation, NELO was present but we failed to detect ILT or ALT. Looking at a publicly available atlas of zebrafish paraffin section stained with H&E (https://bio-atlas.psu.edu/zf/progress.php), we could identify structures reminiscent of NELO in 6 to 7 wpf zebrafish, suggesting that NELO first appears between the fourth and the sixth week of development.

Collectively, our findings from both light and electron microscopy lead us to propose that NELO is a constitutive mucosal lymphoid organ in fish that is associated with the branchial cavity.

### NELO: A lymphoid organ highly enriched with both B and T cells

**Fig. 3.**
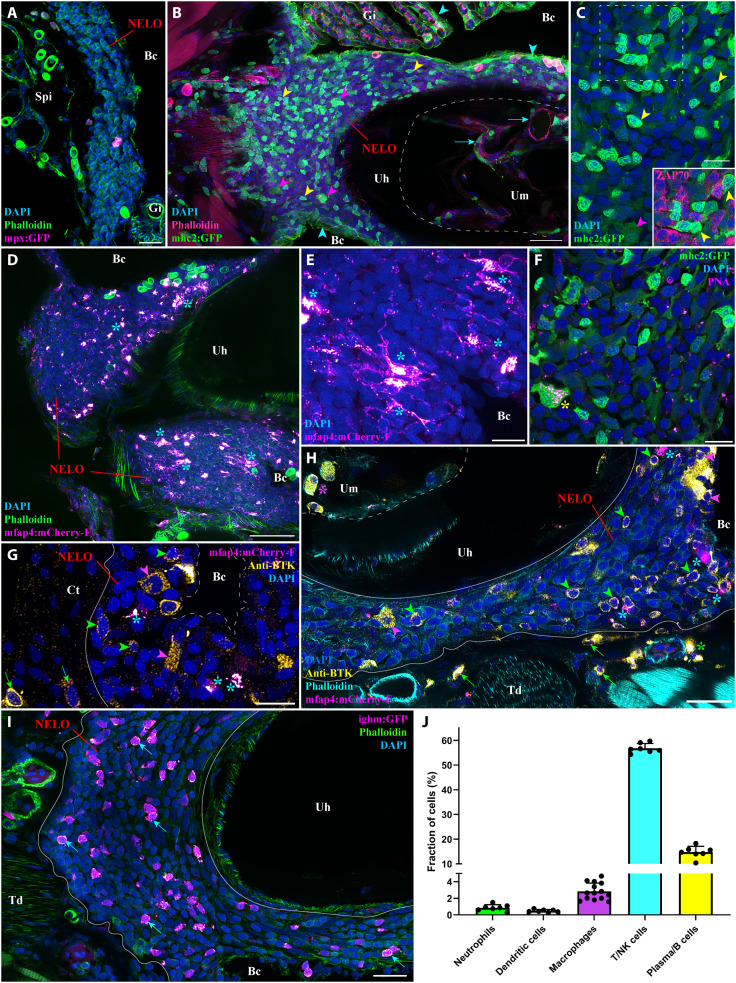
NELO immune cell populations. (**A**) Adult mpx:GFP zebrafish NELO cryosection, in which neutrophils are fluorescent (magenta hot). (**B**) Adult mhc2:GFP zebrafish cryosection, in which mhc2-expressing cells are fluorescent (green). The expression of MHC2 is observed in the epithelial cells (cyan arrowheads), large cells inside NELO (magenta arrowheads), and within the marrow of the urohyal bone (cyan arrows). (**C**) Zoom inside NELO of a mhc2:GFP fish highlighting large (magenta arrowheads) and small (yellow arrowheads) positive cells. The latter being negative for anti-ZAP70 labeling (cherry). (**D** and **E**) Adult mfap4:mCherry-F zebrafish NELO cryosection in which macrophages are fluorescent (magenta hot, cyan stars). (**F**) Cryosection of an adult mhc2:GFP zebrafish NELO stained with peanut agglutinin lectin (magenta hot) to reveal dendritic cell (yellow star). (**G** and **H**) Anti-Bruton’s tyrosine kinase (BTK) labeling (yellow hot) of mfap4:mCherry-F adult zebrafish cryosections revealed macrophages (magenta hot, cyan stars), BTK-positive B cells (green arrowheads) and BTK-positive plasma cells (magenta arrowheads) in NELO. BTK-positive cells were also found in the connective tissue surrounding NELO (green arrows), around endothelial structures (green stars), and within the marrow of the urohyal bone (magenta star). (**I**) The presence of B cells in NELO was confirmed using ighm:GFP zebrafish, in which a subtype of B cells that express immunoglobulin M is fluorescent (magenta hot, cyan arrows). (**J**) Quantification of the different immune cell populations found in NELO counted from at least seven single-cell layer images originating from at least three fish. Annotation: Um, Urohyal marrow. Scale bars, 50 (D), 30 (B), 20 (A, H, and I), and 10 (C and E to G).

Having defined NELO as a mucosal lymphoid organ and described its location in the gill chamber area, the obvious next question was: What role does it play in the fish immune system? We then decided to investigate the diversity of immune cell populations present in NELO in adult zebrafish. Our electron microscopy analysis revealed a small number of neutrophils, identified by their typical elongated granules (Fig. 2, E and H, green). Their presence was confirmed by confocal microscopy using transgenic zebrafish in which neutrophils express fluorescent proteins [*Tg(mpx:GFP)* (*51*)] (Fig. 3A). Also by TEM, fish basophils/mast cells, which displayed characteristic large spherical electron-dense granules, were evident within the connective tissues adjacent to NELO; however, we did not observe any of these cells or eosinophils within NELO itself (Fig. 2E, yellow).

We next investigated the presence of Ag-presenting cells in NELO using *Tg(mhc2dab:GFP)* (*20*) adult zebrafish (Fig. 3, B and C). Consistent with previous studies (*20*, *31*), we observed the expression of the transgene in epithelial cells (cyan arrowheads). Within NELO, the presence of large mhc2^+^ cells (magenta arrowheads) suggested the presence of macrophages and/or dendritic cells. Imaging of *Tg(mfap4:mCherry-F)* zebrafish (*52*), a zebrafish line in which macrophages express a farnesylated membrane-associated fluorescent protein, revealed many large fluorescent macrophages (Fig. 3, D and E, cyan stars). We then labeled dendritic cells using a fluorescent peanut-agglutinin lectin, as described in (*31*, *53*) (Fig. 3E, yellow star). In addition to the large mhc2 cells, we also observed numerous small lymphocyte-like cells in NELO that strongly expressed mhc2 but were negative for ZAP70 (Fig. 3, B and C, yellow arrowheads).

Since B cells are usually known to lack ZAP70 and to express a high amount of MHC2 proteins (*39*, *54*), we hypothesized that these cells could belong to the B cell lineage. We then used a well-characterized antibody against human Bruton’s tyrosine kinase (BTK), which plays a prominent role in B cell biology and development (Fig. 3, G and H) (*55*). Since, in humans, BTK has also been shown to localize in subsets of macrophages (*56*), we used this antibody on mfap4:mCherry-F zebrafish to distinguish cells of the B cell lineage and possible BTK-positive macrophages. The anti-BTK labeling revealed the presence of many B cells (green arrowheads) and plasma cells (magenta arrowheads) within NELO parenchyma, the adjacent connective tissue (green arrows), as well as in the marrow of the urohyal bone (magenta star). Additional data on the anti-BTK labeling are available fig. S7, including the expression of MHC2 by small BTK-positive cells. The identification of B cells in NELO was confirmed by using the *Tg(Cau.Ighv-ighm:EGFP)* transgenic line (Fig. 3I, cyan arrows) (*57*), in which a subset of B cells expressing immunoglobulin M (IgM) was selectively marked. Quantification of the different immune cell types is shown in Fig. 3J and confirmed the predominance of lymphoid cells in NELO: T/NK cells (56.8% of total cells), B cells (14.7%), whereas neutrophils, macrophages, and dendritic cells accounted for less than 5% of the total. The remaining 24% includes the reticulated epithelial cells and the cells forming the squamous epithelial layer. Collectively, these data support our contention that NELO has characteristics predicted for a mucosal secondary lymphoid organ. We emphasize that our analysis does not address all the cell types and cell subsets present in NELO. For this, additional molecular analyses, such as transcriptomics, will be needed.

### Functional characterization of NELO

**Fig. 4.**
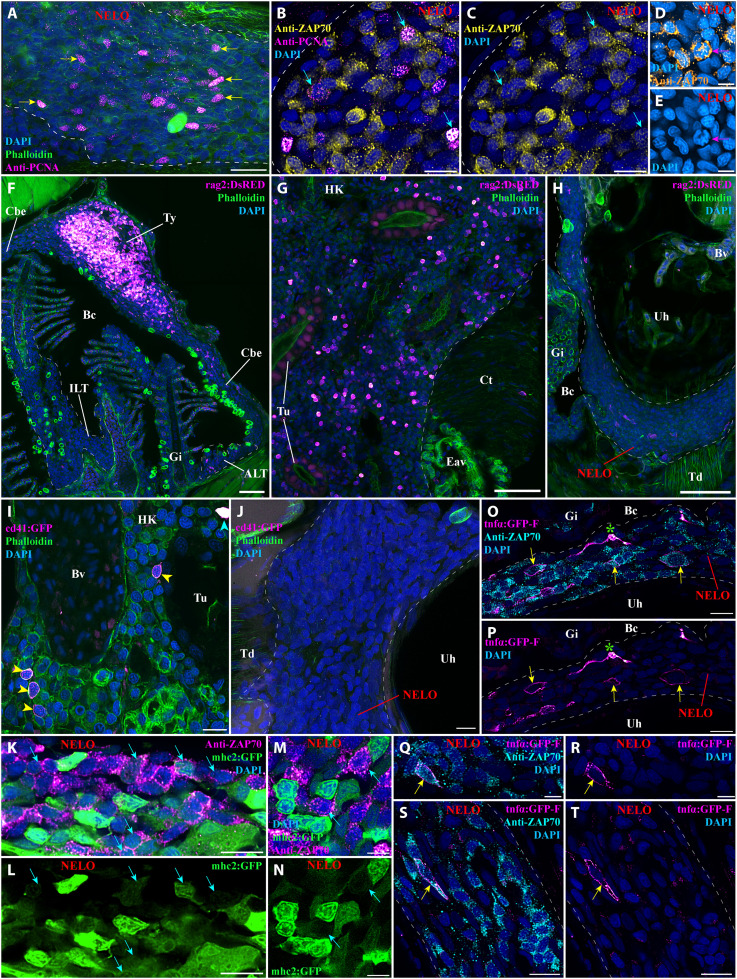
Investigation of immune function molecular markers in NELO. (**A**) NELO cryosection labeled with anti-PCNA antibody (magenta hot) to reveal proliferating cells (yellow arrows). (**B** and **C**) Cryosection co-labeled with anti-PCNA (magenta hot) and anti-ZAP70 (yellow hot) to reveal the presence of proliferative T/NK cells in NELO (cyan arrows). (**D** and **E**) The presence of proliferative T/NK cells in NELO was confirmed by the presence of ZAP70-positive cells (orange hot) displaying mitotic figures (magenta arrow). (**F** to **H**) Cryosections from rag2:DsRED zebrafish in which cells undergoing V(D)J recombination are fluorescent (magenta hot). Whereas numerous positive cells are found in the thymus (F) and the head-kidney (G), which are known sites of V(D)J recombination for T and B cells, almost none were observed in NELO (H), the ILTs and the ALTs (F). (**I** and **J**) Cryosections from cd41:GFP zebrafish, in which thrombocytes (cyan arrowhead) are brightly fluorescent and hematopoietic stem cells are faintly fluorescent (yellow arrowhead) (magenta hot). In contrast to the expected localization of hematopoietic stem cells in the kidney (I), none were observed in NELO (J). (**K** to **N**) Cryosections from mhc2:GFP zebrafish (green) labeled with anti-ZAP70 (magenta hot) revealed the presence of mhc2-expressing T/NK cells (cyan arrows), a feature of activated T/NK cells. (**O** to **T**) Cryosections from tnfα:GFP zebrafish NELO, in which cells expressing the immune effector molecule tumor necrosis factor–α (TNF-α) are fluorescent (magenta hot), labeled with anti-ZAP70 (cyan). Annotations: Bv, blood vessel; Ct, connective tissue; Eav, endothelium anastomotic vessels; HK, head-kidney; Tu, tubule; Ty, thymus. Scale bars, 50 (F to H), 20 (A), 10 (B, C, I to L, O, P, S, and T), and 5 μm (D, E, M, N, Q, and R).

To further investigate the role of NELO, we carried out an initial set of experiments to address whether NELO could be a primary lymphoid organ. We first determined cell proliferation within NELO using a labeling targeting the “proliferating cell nuclear antigen” (PCNA), a protein that is selectively expressed by cells engaged in cell division. As shown in Fig. 4A (yellow arrows), cell proliferation was prominent within NELO, including many PCNA-positive ZAP70-positive cells (Fig. 4, B and C, cyan arrows). Consistent with this result, we observed T/NK cells that displayed mitotic figures (Fig. 4, D and E, magenta arrows). We then investigated the presence of mechanisms involved in the differentiation of lymphoid cells, which are hallmarks of primary lymphoid organs.

The protein RAG2 is an enzyme required for V(D)J recombination that is expressed by developing T cells in the thymus, and by developing B cells in the fish kidney. To determine whether NELO is involved in lymphocyte development, we then used a zebrafish line with a fluorescent reporter for rag2 expression [*Tg(rag2:DsRED*) (*58*)]. In contrast to the typical high expression of RAG2 found in the thymus and the head-kidney, NELO, like the ALT and ILT (*31*, *59*), did not show any notable RAG2 expression (Fig. 4, F to H). This result argues that V(D)J recombination does not take place in NELO, indicating that it is not involved in B and T cell development nor is it an additional thymus.

In adult fish, the production of immune cells by hematopoiesis occurs in the kidney. This process involves hematopoietic stem cells that reside in the immune compartment of the kidney located in between the nephrons. In zebrafish, these cells are identifiable by their low expression of the protein CD41, which in contrast is highly expressed by thrombocytes (fish cells analog of platelets) (*60*). To determine whether NELO represents an additional site of hematopoiesis, we investigated CD41 expression in NELO using the transgenic zebrafish line *Tg(cd41:GFP)*; we could not see hematopoietic GFP expression whereas the hematopoietic stem cells were evident within the kidney (Fig. 4, I and J, yellow arrowheads). Collectively, our data show that NELO is neither involved in lymphocyte V(D)J recombination nor in hematopoiesis, which constitutes strong evidence that it is not a primary lymphoid organ.

We next checked whether NELO displayed features that are characteristic of lymphoid organs involved in immune responses. During our investigation using *Tg(mhc2dab:GFP)* zebrafish, we found ZAP70-positive cells that were also MHC2-positive (Fig. 4, K to N, cyan arrows); this likely indicates the presence of activated T/NK cells in NELO (*61*, *62*). In addition, we also observed ZAP70-positive cells expressing the effector molecule tumor necrosis factor–α (TNF-α )(*63*), using the zebrafish line *Tg(tnfα:eGFP-F)* (Fig. 4, O to T, yellow arrows) (*64*). Together, these results support the concept of NELO being a mucosal secondary lymphoid organ.

### Structural changes in NELO in response to viral and parasitic infection

**Fig. 5.**
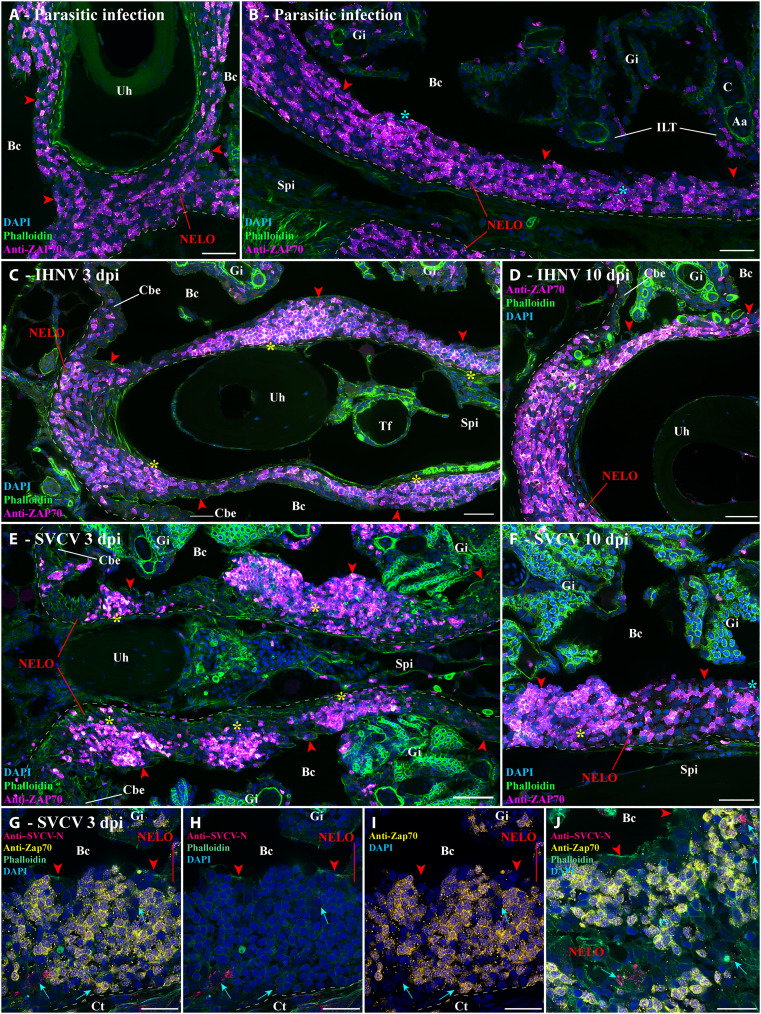
Structural response of NELO to viral and parasitic infections. (**A** and **B**) Cryosections displaying NELO (red arrowheads) in adult zebrafish naturally coinfected with three parasite diseases (*P. neurophilia*, *P. tomentosa*, and *M. streisingeri*) labeled with anti-ZAP70 antibody (magenta hot). The distribution of ZAP70-positive cells in NELO appears more heterogeneous than in uninfected fish. In addition, some the labeled cells formed small clusters (cyan stars). (**C** and **D**) Cryosections from adult zebrafish bath-infected for 24 hours with IHNV. After the apparition of more prominent aggregations of ZAP70-positive cells at 3 dpi (yellow stars) (C), the distribution of T/NK cells reverted to a more homogeneous state by 10 dpi (D). (**E** and **F**) Cryosections from adult zebrafish bath-infected for 24 hours with SVCV. NELO displayed notable aggregations of T/NK cells into distinct clusters at 3 dpi (yellow stars) (E). A week later, NELO displayed both large (yellow star) and small clusters (cyan star) of ZAP70-positive cells (F). (**G** to **J**) Cryosections from zebrafish 3 day after SVCV infection co-labeled with anti-ZAP70 antibody (yellow) and anti-SVCV-N antibody (cherry), revealing cells loaded with viral material (cyan arrows) neighboring large clusters of ZAP70-positive cells. Annotations: dpi, day postinfection; IHNV, infectious hematopoietic necrosis virus; SVCV, spring viremia of carp virus. Scale bars, 50 (E), 30 (A to D and F), and 20 μm (G to J).

If NELO is a mucosal secondary lymphoid organ, it would be expected to be involved in immune responses to infections. Toward this goal, we first investigated zebrafish (*N* = 3) that were naturally coinfected in a zebrafish facility with three different parasites (*Pseudoloma neurophilia*, *Pseudocapillaria tomentosa*, and *Myxidium streisingeri*) that respectively infect the nervous system, the intestines, and the kidneys (*65*). In contrast to uninfected fish (Fig. 1 and fig. S4), the distribution of ZAP-positive cells appeared more heterogeneous, and some of the labeled cells formed small local clusters (cyan stars) (Fig. 5, A and B). Noteworthy, many BTK-positive cells, B cells, and plasma cells were observed within the connective tissue of the sub-pharyngeal isthmus (fig. S8). These changes gave the first hint of a structural rearrangement of NELO in response to long-term parasitic infection. Furthermore, as none of these parasites directly infects the branchial cavity, it also reveals that NELO’s involvement in the immune response is not restricted to the branchial cavity but likely plays a broader function in the overall defense of the organism.

We next studied the effect of controlled bath infection on NELO using two well-established and commercially relevant fish pathogenic rhabdoviruses that infect tissues of the branchial cavity: infectious hematopoietic necrosis virus (IHNV) (*66*) and spring viremia of carp virus (SVCV) (*67*). IHNV infection showed mild structural changes in NELO at 3 days postinfection (dpi) (Fig. 5E, yellow stars) that were evident as a seemingly deeper aggregation of ZAP70-positive cells into large clusters (yellow stars). A week later (10 dpi), these aggregates of cells had reverted toward the usual distribution of ZAP70-positive cells observed in uninfected fish (Fig. 5F). The effect of SVCV infection was more severe with the notable reorganization of ZAP70-positive cells into distinct large clusters at 3 dpi (Fig. 5E and fig. S9, yellow stars). By 10 dpi (Fig. 5F), more ZAP70-positive cells, as well as smaller clusters of labeled cells (cyan star), were observed between the remaining large clusters (yellow star). When we labeled sections from the 3 dpi SVCV-infected fish with an antibody against the N protein of the virus, we detected labeled cells on the periphery of the large T/NK cell clusters (Fig. 5, G to J). Whether these labeled cells represent primarily infected cells or Ag-presenting cells that have taken up viral material remains to be established. Collectively, these data show the involvement of NELO in the organism’s response to viral pathogens infecting tissues of the branchial cavity.

In agreement with our main hypothesis, these results establish the involvement of NELO in immune responses. However, while our study provides a solid foundation to study NELO’s involvement during infection, further research is required to strengthen our understanding of NELO’s contribution to the teleost immune response.

### NELO, the ILTs, and ALTs as a cohesive unit of a vast lymphoid network inside the branchial cavity

Our next objective was to further investigate NELO in the context of the branchial cavity. NELO was intimately connected to the eight ILTs and 16 ALTs (Fig. 1 and fig. S4) with whom it shares the same network of reticulated epithelial cells (fig. S5A, red stars, and movie S7), indicating that NELO, the ALTs, and the ILTs form a cohesive unit within the branchial cavity (Fig. 1). To better appreciate the relation between NELO and the lymphoid aggregates, we also looked at the structural response of the ILTs to infections. In parasite-infected fish, both ILTs and NELO displayed a similar structural response as described above (fig. S10A). In both SVCV-infected and IHNV-infected fish, however, the ILTs were strongly reduced while NELO persisted (fig. S10, B and C) (*31*)at 3 dpi, suggesting that NELO and gill lymphoid aggregates may play a different role in cellular responses to viral infections.

**Fig. 6.**
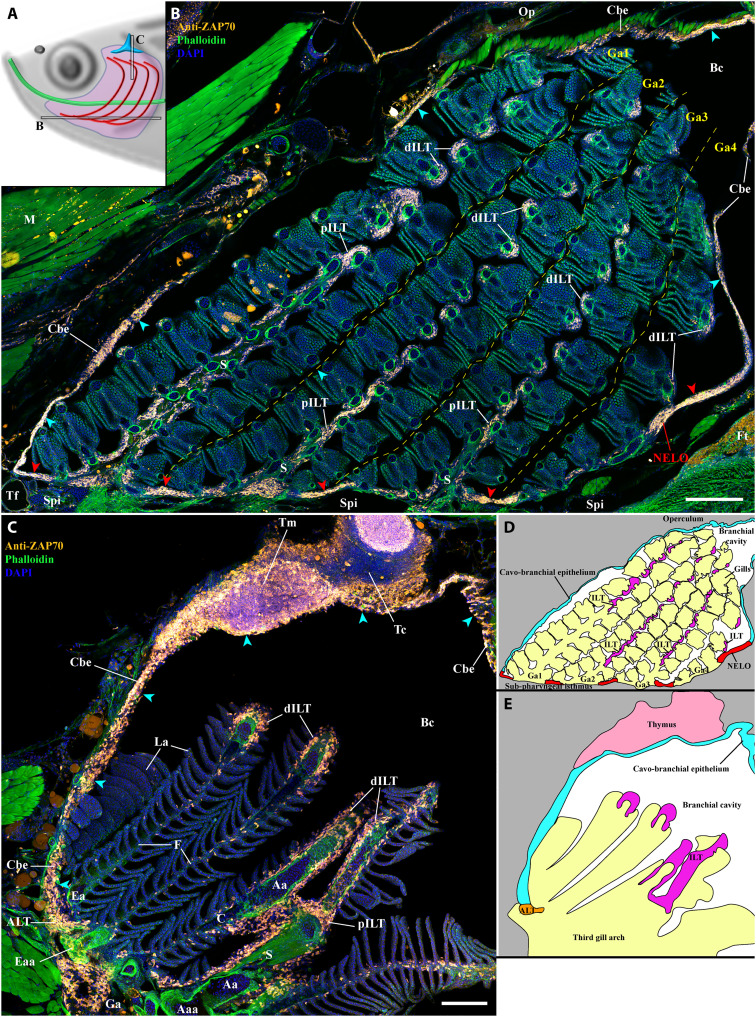
NELO as part of a larger lymphoid network. (**A**) Scheme illustrating the localization of the branchial cavity images acquired from 30-μm whole adult zebrafish head cryosections labeled with anti-ZAP70 antibody (orange hot) to reveal T/NK cells (**B** and **C**). NELO (red arrowheads) is part of the lymphoid network propagated by the ZAP70-positive cell–rich cavo-branchial epithelium (cyan arrowheads) and which connects all the lymphoid structures of the branchial cavity. (**D**) Scheme describing the different anatomical regions displayed by the coronal cryosection presented in (B). (**E**) Scheme describing the different anatomical regions displayed by the transversal cryosection presented in (C). Annotations: Aaa, afferent arch artery; dILT, distal interbranchial lymphoid tissue; Eaa, efferent arch artery; Ft, fat tissue; Op, operculum; pILT, proximal interbranchial lymphoid tissue; Tc, thymus cortex; Tm, thymus medulla. Scale bars, 200 (B) and 100 μm (C).

In addition to the ILT and ALTs, NELO was also in continuity with regions of the cavo-branchial epithelium. These regions of junctions contained numerous T/NK cells (Fig. 1B, cyan arrowheads). In fact, most of the cavo-branchial epithelium (Fig. 6, B and C, cyan arrowheads) encompassed a high number of ZAP70-positive cells, forming a vast lymphoid network within the branchial cavity that links NELO, the sixteen ALTs, the eight ILTs, and even the two thymus lobes. Further analysis showed that this lymphoid network extended beyond the branchial cavity region (fig. S11), likely connecting with the tessellated lymphoid network (*42*) via the operculum opening, as well as linking the pharyngeal epithelium and the esophagus epithelium (fig. S11A). This lymphoid network further extended along the pharynx and the mouth (fig. S11, B and C), from which it connected with the SALT via the nonkeratinized sides of the mouth opening (fig. S11D). In line with a previous study describing teleost fish SALT (*29*), it then connected with the NALT via a skin network of T/NK cells located in the basal layers of the epidermis and surrounding club cells (fig. S11, E to G, and movie S8). Noteworthy, localized clusters of ZAP70-positive cells were observed in the epidermis of the zebrafish head, which may represent localized structured units of the SALT (fig. S11, C, D, and G, green stars).

### NELO and its cohesion with ILTs and ALTs in other teleost fish species

**Fig. 7.**
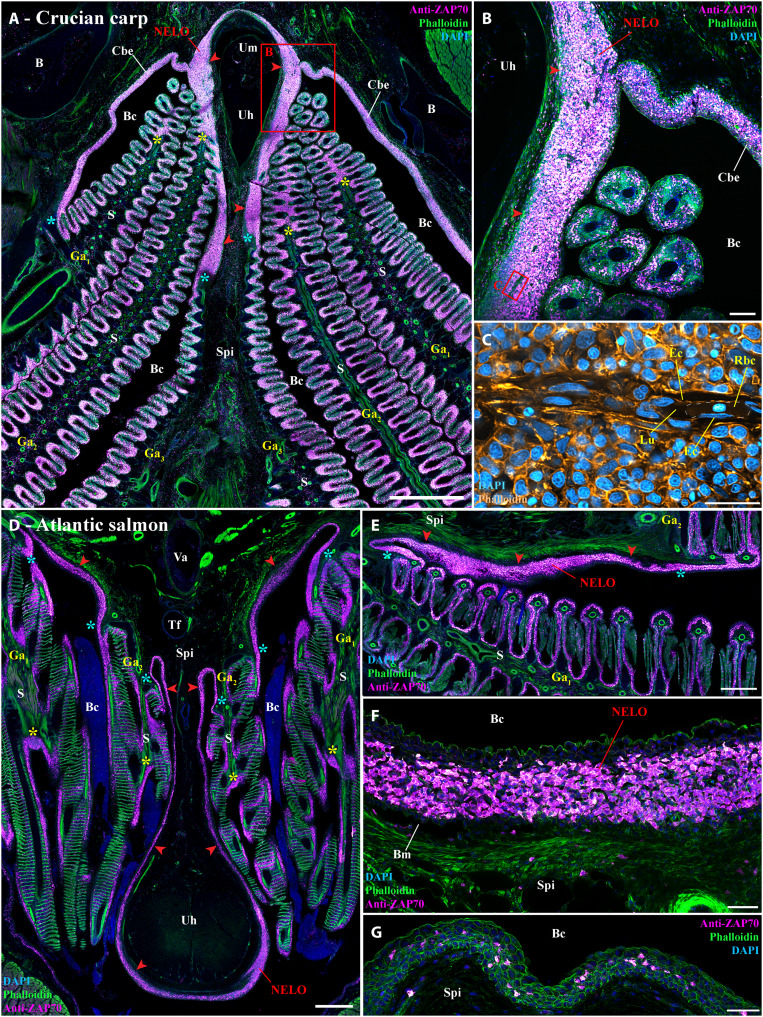
NELO and its cohesion with ILTs and ALTs in other teleost fish species. (**A** and **B**) Cryosection from wild crucian carp labeled with anti-ZAP70 antibody (magenta hot). This larger representative of the cyprinids than zebrafish also has NELO (red arrowheads), ILTs (yellow stars), and ALTs (cyan stars), which are all interconnected by a clear lymphoid network. (**C**) Zoom from (B) in which phalloidin (orange hot) and DAPI (cyan hot) staining revealed the presence of endothelial vessels containing red blood cells. Transversal (**D**) and coronal (**E**) cryosections of an adult Atlantic salmon (1.5 kg) labeled with anti-ZAP70 (magenta hot) displaying NELO (red arrowheads), ILTs (yellow stars), and ALTs (cyan stars). As for cyprinids, NELO (red arrowheads) of this representative of the salmonid family is located at the ventral convergence of gill arches, interconnected with gills lymphoid aggregates, and closely associated with the urohyal bone. It is particularly enriched in ZAP70-positive cells (**F**). In contrast, the mucosal epithelium lining the region directly anterior to the urohyal bone only displayed scarce T/NK cell (G). Annotations: B, bone; Ec, endothelial cell; Lu, lumen; Rbc, red blood cell. Scale bars, 1000 (A), 500 (D), 400 (E), 100 (B), 50 (F and G), and 20 μm (C).

Our next objective was then to determine whether NELO exists in other fish species. Since the zebrafish is a small representative of the cyprinid fish family, we first asked whether a larger cyprinid would share the same branchial cavity lymphoid organization. For this, we labeled wild crucian carp (*Carassius carassius*) with the anti-ZAP70 antibody, which revealed a lymphoid organization that was notably similar to the zebrafish (Fig. 7, A to C). As in zebrafish, crucian carp NELO formed an important mass of T/NK cells along the sub-pharyngeal isthmus in close association with the ILTs, the ALTs, and the ZAP70-positive cell–rich mucosal epithelium lining the branchial cavity (Fig. 7, A and B). Whereas zebrafish NELO was not infiltrated by endothelial vessels, NELO of crucian carp contained clear endothelial structures in which red blood cells could be observed (Fig. 7, B and C).

We then extended our investigation to the adult Atlantic salmon (*S. salar*), a representative salmonid (Fig. 7, D to G). While adult Atlantic salmon is anatomically divergent from zebrafish and crucian carp (e.g., open branchiostegal rays), and displays different adaptations (e.g., Atlantic salmon is an anadromous marine fish), we found a structure in salmon that was very similar to cyprinid NELO: It was also wrapped around the urohyal bone and intimately connected to the gill lymphoid aggregates (Fig. 7, D and E). In contrast to the abundance of T/NK cells in Atlantic salmon NELO (Fig. 7F), fewer ZAP70-positive cells were observed in the mucosal epithelium anterior to the urohyal bone (Fig. 7G). The existence of the Atlantic salmon NELO was confirmed using an anti-CD3ɛ monoclonal antibody, which specifically labels T cells (fig. S12). This immunolabeling suggests that most ZAP70-positive cells in NELO and the gill lymphoid aggregates are likely CD3-positive T cells rather than NK cells. We could also recognize a structure reminiscent of NELO in three-spined stickleback (*Gasterosteus aculeatus*), a representative of the family gasterosteids (*68*), which belongs to the most derived clade of euteleost, the percomorphs.

Collectively, this probing comparative study indicates that NELO and the branchial cavity lymphoid organization we describe here in zebrafish are conserved among members of the cyprinid and salmonid families, the two most extensively studied and farmed families worldwide, as well as in (at least) some percomorphs. Furthermore, our data suggest that cohesiveness with gill lymphoid aggregates and the anatomical associations with the urohyal bone and sub-pharyngeal isthmus might represent typical features of NELO across different teleost species.

## DISCUSSION

In the present study, we investigated the structural organization of lymphoid tissues in the zebrafish branchial cavity. We show that the tissues lining the branchial cavity, which are highly exposed to the pathogens of the aquatic environment, present a complex network of connected immune tissues. Advanced imaging of this region led us to identify a previously unknown mucosal lymphoid organ, which we tentatively named the Nemausean lymphoid organ (NELO), that is associated with the fish pharyngo-respiratory tract. Detailed investigations of its structural organization by light and electron microscopy provided a solid foundation to characterize NELO as a lymphoid organ, such as the presence of an intricate network of reticulated epithelial cells. NELO was a prominent constituent of a cohesive unit, formed with ILTs and ALTs, of a lymphoid network interconnecting all the lymphoid structures in the branchial cavity. This highlights a higher level of integration of the surveillance and defense system associated with the fish pharyngo-respiratory tract. Given the central localization of NELO within the branchial cavity, we hypothesized that NELO could constitute a secondary lymphoid organ in which immune responses would occur. This idea was supported by the characterization of its immune cell populations that showed a high enrichment in T/NK cells and B cells mixed with Ag-presenting cells and by the lack of expression of the recombinase required for V(D)J rearrangements, RAG2. NELO also contained ZAP70^+^ PCNA^+^ proliferating T cells, and ZAP70^+^ MHCII^+^ and ZAP70^+^ TNF-α^+^ activated T cells, further strengthening the hypothesis that it is a secondary lymphoid organ. Following infection by different pathogens, NELO underwent structural changes involving the formation of ZAP70^+^ cell clusters, suggesting direct involvement in immune responses.

During our investigations, we could make an initial appreciation of NELO’s ontology using our data and the zebrafish histology atlas (https://bio-atlas.psu.edu/zf/progress.php) and found that NELO appears unexpectedly late during development. Whereas the main lymphoid organs (thymus, spleen, and kidney) are already present by the first 2 weeks of the zebrafish development (*69*, *70*), NELO likely appears around the fourth to sixth weeks of development, right after the larval-juvenile transition stage. Thus, NELO’s development coincides with the emergence of fully mature adaptive immunity (*70*, *71*). The gill lymphoid aggregates (ALT and ILT), to which NELO is tightly connected, also appear during the same time window, or possibly just after the development of NELO. The appearance of these lymphoid structures is particularly notable when one considers that 3 wpf zebrafish contain very few T/NK cells, aside from those in the thymus lobes. Further work will be required to understand the developmental mechanisms involved in this drastic transformation of the immune landscape of the branchial cavity and its impact on the fish’s susceptibility to infections. It is well known that fish fry are highly sensitive to many viral and bacterial infections compared to adults. In mammals, thyroid hormones have a serious impact on lymphoid organs’ development and biology (*72*). For example, early removal of the thyroid in rats alters lymphoid organ morphology and impairs function (*73*). In zebrafish, thyroid hormones are important for the larval-juvenile transition stage, and they also influence the size of the thymus (*71*, *74*, *75*). The possibility that thyroid hormones may affect NELO’s development, and more generally, the relocation of the T cells into the branchial cavity, would deserve some attention. The presence of a recently reported zebrafish cell population sharing molecular markers with mammalian lymphoid tissue inducer cells (*17*), a type of innate lymphoid cell that is involved in the formation of certain secondary lymphoid organs in mammals, should also be investigated in NELO and in the branchial cavity at the end of the larval-juvenile transition stage.

An interesting question is whether NELO has counterparts among vertebrates or if this organ represents an independent solution developed by certain fish species to combat infectious diseases. The evolutionary history of lymphoid organs has been investigated and intensely debated using various terminologies and classification criteria (*3*–*6*, *8*–*10*). In addition to the thymus, the presence of lymphoid aggregates associated with the pharyngeal region has been reported in mammals, birds, reptilians, and amphibians, but so far not in teleostean or chondrostean fish. Over a century ago, Salkind (*8*) mentioned the existence of lymphoid concentrations within the lower pharynx of Brook lamprey (*Lampetra planeri*), an agnathan; he considered that these structures were distinct from the presumptive thymus. The absence of prior recognition of a prominent pharyngeal lymphoid structure such as NELO underscores that teleost fish lymphoid organization remains incompletely documented. Collectively, a fascinating question that arises from this work is whether the formation of mucosal secondary lymphoid structures within the pharyngo-respiratory region is a conserved developmental program shared by vertebrates.

In mammals, the nasopharyngeal area is protected by a set of mucosal secondary lymphoid organs called tonsils, which collectively form Waldeyer’s tonsillar ring (*76*). Although our work does not provide evidence that NELO is homologous to mammalian tonsils, it supports the hypothesis that NELO may represent their fish analog. NELO, like the tonsils, is a mucosal lymphoid organ located within the pharyngo-respiratory tract, which is exposed to the external environment. As with tonsils, NELO constitutes a mass of lymphoid cells structured by an intricate network of reticulated epithelial cells. Moreover, in certain fish species, NELO exhibits clear signs of vascularization. NELO is part of a larger lymphoid network containing 25 lymphoid structures (8 ILTs, 16 ALTs, and NELO) that are strategically positioned within the pharyngo-respiratory region at sites exposed to Ags encountered during feeding and breathing. This arrangement might represent in fish a distant functional analog of Waldeyer’s ring of tonsils. Last, as palatine tonsils, NELO appears relatively late in development at a site associated with the second pharyngeal pouch (*77*–*79*). However, mammalian tonsils display a more complex structural organization than NELO, such as well-defined T cell and B cell zones. Similarly, the presence of germinal center-like structures in NELO remains to be explored.

The analysis of NELO cellular composition is particularly relevant for our understanding of teleost fish immunology. We showed that NELO contains more T/NK cells than the spleen, which is considered the primordial secondary lymphoid organ in fish (*4*), suggesting that it may play an essential role in the homeostasis of adaptive immunity. Ablation of NELO or T cell depletion experiments, for example, using rag2 mutant zebrafish (*80*), could provide valuable information on NELO’s function. In addition, we showed that NELO also has a substantial plasma/B cell population. Secretory antibodies play an important role in the maintenance of the branchial cavity homeostasis, as shown by Xu *et al.* (*81*), the experimental depletion of secretory IgT in rainbow trout induced gill dysbiosis, inflammation, and tissue damage. Further studies would have to clarify the role of NELO in immune system regulation and the screening of external pathogens from local microbiota.

The infection experiments we performed have been key to proposing NELO as a secondary lymphoid organ. After 3 days of infection by SVCV, NELO displayed a rearrangement of T/NK cells into large clusters surrounded by cells carrying virus material. Further studies would have to determine the nature of these concentrations of lymphoid cells we observed in NELO and if they represent structures favoring processes of adaptive immunity such as Ag presentation. It would be particularly interesting to determine whether they are associated with the formation of melanomacrophage centers, which are immune structures that have been suggested as potential analogs of mammalian germinal centers in teleost fish (*82*) and are occasionally observed in NELO of Atlantic salmon (fig. S12, cyan arrowheads) and zebrafish (fig. S13).

Our observations revealed a structural cohesion between NELO, ALT, and ILTs, all sharing the same network of reticulated epithelial cells. The ILT was the first structured mucosal lymphoid tissue found in fish. Studies in salmonids showed that it may represent a nonconventional secondary lymphoid tissue (*25*, *59*). This was followed by our description of the ALT in zebrafish. Consistent with studies in salmon, the zebrafish ILT and ALT displayed features of secondary lymphoid organs. In this study, we showed that ILTs and ALTs are not just a set of 24 distinct lymphoid structures, they are bound together by a more prominent lymphoid organ that also has features of secondary lymphoid organs, NELO. NELO, the ALTs, and ILTs did not display a similar structural response to IHNV and SVCV infections in adult zebrafish (*31*). The zebrafish ILT and ALT are first strongly reduced by 3 dpi, which is consistent with a reduction of the ILT described in Atlantic salmon infected with infectious salmon anemia virus (*59*), whereas NELO persisted. This constitutes a major difference between NELO and the gill lymphoid aggregates, which indicate that they likely bear different immune functions. However, NELO and the ILT displayed a similar rearrangement of ZAP70 cells in the fish naturally coinfected with multiple parasites. Further studies would have to determine whether the gill lymphoid aggregates are constituents of NELO.

Our investigations of the whole branchial cavity revealed the presence of a vast lymphoid network that links NELO, ILTs, ALTs, and thymus lobes together into a sophisticated superstructure of the fish immune system, which suggests the branchial cavity may act as a lymphoid nexus. The implications of this lymphoid superstructure for the homeostasis of the fish immune system and its interactions with other tissues/organs remain to be deciphered.

In this study, we recognized NELO in representatives of distant teleost fish species families: two cyprinids, one salmonid, and a Percomorph. This observation suggests that NELO may be present in most fish families. We anticipate a substantial level of structural variability in NELO across teleost fish species, given the taxonomic and morphologic diversity within this group. However, our data suggest that its position around the urohyal bone and at the convergence of gill arches along the sub-pharyngeal isthmus is a conserved feature. As in zebrafish, the integral unit formed by NELO and the gill lymphoid aggregates was clear in all the analyzed fish species, suggesting that the NELO/ALT/ILT apparent unity might be an essential arrangement for immune responses in the branchial cavity. Taking into account our previous comparative studies, ILTs seem to be only present within the gills of “basal” teleost, whereas ALT and NELO could be observed in all the analyzed teleost fish species so far. It would be of particular interest to investigate whether the morphology of the branchial cavity and, more specifically, the evolution of the branchiostegal rays (*83*) influence the existence or the morphology of NELO and its unity with gill lymphoid aggregates.

Together, our study provides new insights into the teleost fish immune system and its structural organization. We identified an unreported lymphoid organ within the pharyngo-respiratory region of adult zebrafish and other teleost species, which we named the Nemausean lymphoid organ (NELO). Our investigations led to the idea that NELO is a fish mucosal secondary lymphoid organ that shows many features resembling of mammalian tonsils. Intimately associated with gill lymphoid aggregates, NELO appears as a potential key lymphoid hub coordinating lymphocyte traffic and defense mechanisms within the fish respiratory mucosa. Collectively, our findings contribute to a better understanding of the evolution of the vertebrate immune system and provide new insights into fish immunology. Gill immunity is of growing importance both for future aquaculture vaccines and for the development of zebrafish disease models.

## MATERIALS AND METHODS

### Animal care and ethic statement

Experiments were conducted in compliance with the animal care guidelines, ethical standards, and legislation of the European Union, France, and Norway, and in consultation with local ethics committees. Animal experiments performed in the present study were carried out at the IERP fish facilities (building agreement no. C78-720, doi.org/10.15454/1.5572427140471238E12) of the INRAE Research Center at Jouy-en-Josas, France, in compliance with the recommendations of Directive 2010-63-EU on the protection of animals used for scientific purposes. These infection protocols were authorized by the institutional review ethics committee, COMETHEA, of the INRAE Research Center. Authorizations were approved by the French Ministry of Agriculture (authorization number APAFIS# 22150-2019062811528052).

Animal experimentation, handling, and euthanasia were performed by well-trained and authorized staff. Specimen were euthanized using an anesthetic overdose of buffered tricaine.

The experiments were performed using AB wild-type zebrafish (around 1 year, unless specified) (*N* = 36) and the following transgenic lines: *Tg(lck:EGFP)* (*N* = 3) (*18*), *Tg(fli1a:EGFP)^y1^* (*N* = 3) (*46*), *Tg(mpx:GFP)^i114^* (*N* = 3) (*51*), *Tg(mhc2dab:GFP)^sd6^* (*N* = 3) (*20*), *Tg(mfap4:mCherry-F)^ump6^* (*N* = *6*) (*52*), *Tg(Cau.Ighv-ighm:EGFP)^sd19^* (*N* = *3*) (*57*), *Tg(rag2:DsRED*) (*N* = 3) (*58*), *Tg(cd41:GFP)* (*N* = 3) (*60*), and *Tg(tnf*α*:eGFP-F)^ump5^* (*N* = 3) (*64*).

The study includes three laboratory-grown adult zebrafish (1 year) naturally coinfected with *P. neurophilia*, *P. tomentosa*, and *M. streisingeri*, provided by the Oehlers’ laboratory (Singapore). The fishes were held at the IMCB Zebrafish facility under A*STAR IACUC approval 211667 and were sampled as part of a culling following veterinarian diagnosis. Animal handling and euthanasia were performed in accordance with Singaporean regulations.

Two healthy adult Atlantic salmon (weight: 1500 g), laboratory-raised by NIVA (Solbergstrand, Norway), were provided by PHARMAQ, a division of Zoetis. The fish were handled and euthanized in strict accordance with Norwegian legislation by authorized staff.

Three wild crucian carps (40 g, both sex), captured using a nylon net in October 2020 in Tjernsrud pond (Oslo, Norway), with a healthy appearance upon sampling were provided by the Lefevre-Nilsson group from University of Oslo. Specimen were sampled and euthanized in compliance with Norwegian animal welfare laws (Dyrevelferdsloven), carried out as part of the authorized project FOTS permit ID 16063, and following the instruction about the use of animals for research (Forskriften om bruk av. dyr I forsøk).

### Infection experiments with SVCV and IHNV

SVCV and IHNV infectious challenges were carried out on wild-type zebrafish of the AB strain, aged 16 months, and weighing 0.8 g (±0.03 g). Fish were acclimatized for 48 hours at 22°C (pH 7; conductivity, 200 μS/cm^2^) in 1.5-liter aquaria. Two groups of eight fish each were then infected by immersion for 48 hours using the reference SVCV strain VR-1390 (*84*) and the IHNV strain 25-70 adapted to 25°C (*85*), at a final concentration of 10^4^ plaque-forming units/ml. The water flow was stopped for 48 hours, followed by daily water change. Fish were euthanized and sampled at 3 and 10 dpi by IHNV, and at 3 and 10 dpi by SVCV. Noninfected controls were prepared in parallel (*n* = 4).

### Electron microscopy

Juvenile zebrafish (9 wpf, *N* = 3) were euthanized and immediately immersed in 20 ml of fixative [4% formaldehyde, 0.8% glutaraldehyde, in 1× PHEM buffer (*86*, *87*) (pH7.2) in fish water] for 24 hours at room temperature (RT), followed by a 24-hour incubation at 4°C. Samples were quenched in 100 mM glycine for 2 hours at RT and rinsed with 100 mM sodium bicarbonate buffer (pH 6.5). For postfixation, samples were incubated on ice with a solution of 2% osmium tetroxide and 1.5% potassium ferricyanide in 100 mM sodium bicarbonate buffer (*88*), rinsed five times with 100 mM sodium bicarbonate buffer and two times with 50 mM maleate buffer (pH 5.15), and incubated in 2% uranyl acetate in 50 mM maleate buffer for 3 hours. Following washes in 50 mM maleate buffer, gradual dehydration was achieved by “progressive lowering of temperature” using the following sequential incubations: 1 hour 30% ethanol on ice, 1 hour 50% ethanol on ice, 30 min 1% uranyl acetate in 70% ethanol at −20°C, 1 hour 70% ethanol at −20°C, 90 min 80% ethanol at −30°C, 90 min 90% ethanol at −30°C, 2 hours 96% ethanol at −30°C, 16 hours 100% ethanol at −30°C, three times 2 hours 100% dry ethanol (3-Å molecular sieve) at −30°C, two times 30 min dry acetone at −30°C, and 14 hours 25% EPON in dry acetone at RT. EPON was prepared with a ratio of 3:7 (dodecenylsuccinic anhydride:nadic methyl anhydride) containing 1% 2,4,6-Tri(dimethylaminomethyl) phenol (DMP-30). Specimen infiltration was done by a 24-hour incubation in 100% EPON at RT. Samples were then embedded in fresh EPON using flat embedding molds and oriented after 3 hours of polymerization at 60°C. Last, polymerization was performed for 48 hours at 60°C followed by a 24-hour curing period at RT. Targeted trimming was aided by staining semithin (300 nm) sections with 0.1% toluidine blue in borate buffer (pH 11) at 80°C to facilitate the orientation in the sample. Samples were sectioned at 60-nm thickness on a Leica UCT ultramicrotome using Diatome 45° ultra knives and mounted on carbon-coated, formvar film on 2-mm single-hole copper grids. Sections were then stained with 4% uranyl acetate in 50% methanol for 1 hour, followed by a 20-s incubation with Reynolds lead citrate. Images were acquired at 120 kV with a Jeol JEM-1400 electron microscope using a Tvips 216 camera. The manually recorded images were aligned using the plugin Big Stitcher in ImageJ, montaged using GIMP for layer projection, and colored using Photoshop CS6. If applicable, the generation of a tile pyramid and the visualization via Java were done using OpenSeadragon and OpenLayers on a basic HTML site.

Ultrastructure maps of NELO are available on figshare.com (DOI: 10.6084/m9.figshare.22259698) and on https://wohlmann.github.io/2019019_004_M1c/ and https://wohlmann.github.io/2019019_004_N2/.

### 3D reconstruction of the zebrafish NELO

Following euthanasia, adult zebrafish heads (15 wpf, *N* = 3) were fixed in a solution of 4% methanol-free formaldehyde (Thermo Fisher Scientific) in 60 mM Hepes buffer (pH 7.4) for 24 hours at RT, followed by a 3-day incubation at 4°C. For decalcification, samples were incubated in a solution of 13% EDTA (pH 7.8), 0.1% Tween 20 (Sigma-Aldrich), and 1% Triton X-100 (Sigma-Aldrich), in ddH_2_O for 5 days at RT under gentle rocking. Samples were then saturated for 24 hours at RT in a BlockAid solution (Thermo Fisher Scientific) with 0.5% Triton X-100 and 0.1% Tween 20. T/NK lymphocytes were labeled using a rabbit anti-ZAP70 monoclonal antibody (99F2, Cell Signaling Technology) diluted at 1:600 in Pierce Immunostain Enhancer solution (Thermo Fisher Scientific) complemented with 0.5% Triton X-100 and 0.1% Tween 20 for 5 days at RT under gentle rocking. Samples were then washed several times at RT in 1× PHEM buffer [60 mM Pipes, 25 mM Hepes, 10 mM EGTA, and 2 mM MgCl2 in ddH_2_O (pH 7.4) (*86*, *87*)] with 0.5% triton X-100 and 0.1% Tween 20 (PHEM_t-tw_), and incubated with goat anti-rabbit Alexa Flour 647 (Jackson ImmunoResearch) diluted at 1:400 in 1× PHEM_t-tw_, complemented with phalloidin–tetramethyl rhodamine isothiocyanate (TRITC) (Sigma-Aldrich) at 3 U/ml and DAPI (Thermo Fisher Scientific) at 5 μg/ml, for 5 days at RT under gentle rocking. Samples were then first rinsed with 1× PHEM_t-tw_ and then 1× PHEM. Samples were stored at 4°C in 1× PHEM until further processing. One sample was then sliced and mounted onto a coverslip with SlowFade glass mounting medium (Thermo Fisher Scientific) to control the quality of the labeling and for wholemount imaging of the skin covering the head.

Tomography was performed using an automatized Zeiss LSM 880 confocal microscope coupled with a vibratome (Microm HM 650 V from Thermo Fisher Scientific). For sectioning, ZAP70-labeled zebrafish heads were embedded in 6% agarose in water within a 1-cm^2^ plastic chamber and orientated for appropriate cross-sectioning with the rostral side on top. Once set, the agarose box was resized using a razor blade and was attached with superglue on a metal surface with the rostral side orientated on top and placed into a tank filled with water. The following automated process was then applied: an 80-μm-thick layer was removed from the surface of the block containing the samples by the vibratome, followed by the immediate imaging of the newly exposed surface with the Zeiss LSM 880 confocal microscope. Images were acquired in confocal mode, with a 20× Plan Apo 1.0 NA water immersion objective, a wavelength of 633 nm for excitation and 660- to 711-nm band for emission and a wavelength of 561 nm (Argon Laser) and 561- to 630-nm band (GaAsP detector), sequential mode, a mosaic of 12 × 12 fields, a stack of 102-μm total volume, and 6-μm steps. Imaging steps were repeated and 3D files generated from acquisitions were processed using ImageJ for alignment, stitching, and cropping. NELO, the thymus lobes, the ALT, and the ventral end of the gill arches were manually segmented on each single layer based on the phalloidin and anti-ZAP70 signals using Imaris to assemble the different 3D reconstructions. IMARIS was also used to generate the 3D videos.

### Immunofluorescence: Cryosections

Following euthanasia, whole adult zebrafish and dissected lower pharyngeal areas of both Atlantic salmon and crucian carp were fixed in a solution of 4% methanol-free formaldehyde (Thermo Fisher Scientific) in 60 mM Hepes buffer (pH 7.4) for 24 hours at RT, followed by a 3-day incubation at 4°C. Atlantic salmon and crucian carp samples were decalcified with a 5-day incubation in a solution of 13% EDTA (pH 7.8) in ddH_2_O at RT under gentle rocking.

Samples were cryoprotected by two incubations in a solution of sucrose at 32% in ddH_2_O until the specimens sunk to the bottom of the solution and then were embedded in Tissue-Tek O.C.T. Compound (Sakura Finetek USA, Mountain View, CA, USA). Samples were flash-frozen in isopentane and sectioned using a CM1950 cryostat (Leica, Wetzlar, Germany). The resulting 30-μm cryosections were collected on Superfrost Plus slides (Thermo Fisher Scientific) and stored at −20°C. Samples used for stereology analyses were sectioned and recovered in a standardized uniform random way.

Following the protocols detailed in (*31*), immunofluorescence is as follows. Briefly, following saturation in BlockAid solution (Thermo Fisher Scientific), slides were incubated with one or several of the following primary antibody/lectin: 1:300 rabbit anti-ZAP70 monoclonal antibody (99F2, Cell Signaling Technology), 1:40 Cytokeratin Pan Type I/II mouse monoclonal antibody cocktail (Thermo Fisher Scientific), 1:300 mouse anti-PCNA monoclonal antibody (PC10, Thermo Fisher Scientific), 1:200 mouse anti-BTK monoclonal antibody (D6T2C, Cell Signaling Technology), mouse anti-CD3ɛ monoclonal antibody (5 μg/ml) cross-reacting with Atlantic salmon (*89*), and 1:20 mouse anti-SVCV-N monoclonal antibody (BIO 331, Bio-X Diagnostics). Peanut agglutinin lectin coupled with Alexa Fluor 594 (1:200; Thermo Fisher Scientific). When necessary, sections were incubated with one or several of the following cross-adsorbed secondary antibodies at 1:250: Goat anti-rabbit Alexa Fluor 647 (Jackson ImmunoResearch), Goat anti-mouse Alexa Fluor 647 (Jackson ImmunoResearch), and Goat anti-mouse Alexa Fluor 594 (Jackson ImmunoResearch). Where relevant, secondary antibodies or lectin were coincubated with fluorescent phalloidin (TRITC- or fluorescein isothiocyanate–labeled, Sigma-Aldrich) at 3 U/ml, and DAPI (Thermo Fisher Scientific) at 5 μg/ml. Slides were mounted with coverslips using ProLong glass mounting medium (Thermo Fisher Scientific).

### Imaging and image analysis

Three-dimensional images were acquired with the Zyla camera of a Dragonfly 500 spinning disk confocal microscope (Andor, Belfast, UK), with 40-μm pinholes and either a 20×/0.75 dry objective or a 60×/1.4 oil-immersion objective. Acquisitions, stitches, and deconvolutions (14 to 16 iterations) were performed using the built-in features of the Fusion software. Image analyses were carried out using IMARIS and ImageJ/Fiji software. The acquisition and analyses of images were made at the NorMIC imaging platform (University of Oslo, Norway).

The average volume of a zebrafish T/NK cell was quantified using IMARIS by reconstructing the 3D structure of 15 random ZAP70-positive cells from 3D images of NELO cryosections coming from three different fish.

The volume of NELO, and spleen, occupied by ZAP70-positive cells was calculated with “point counting stereology” (*90*) using ImageJ to generate randomly placed 500- and 100-μm^2^ grids on single optical section images of the anterior segment of NELO collected from three different fish. The amount of T/NK cells was then calculated by multiplying the total volume of the organ by the fraction of the volume occupied by ZAP70-positive cells, which was then divided by the average volume of a single T/NK cell.

Cell counting was performed manually using the “Cell counter” plugin on ImageJ on single optical section images of the anterior segment of NELO coming from three different fish. Graphs were generated using the software GraphPad Prism 7.
