# Supplementary material for: Identification of a pharyngeal mucosal lymphoid organ in zebrafish and other teleosts: Tonsils in fish?
Source: Sci Adv. 2023 Nov 1;9(44):eadj0101. doi: 10.1126/sciadv.adj0101 (PMC10619939; doi:10.1126/sciadv.adj0101)
Supplement: Supplementary file 1 — Figs. S1 to S13 Legends for movies S1 to S8 [file sciadv.adj0101_sm.pdf]

Science Advances
AAAS

Supplementary Materials for

Identification of a pharyngeal mucosal lymphoid organ in zebrafish and other teleosts: Tonsils in fish?

Julien Resseguier *et al.*

Corresponding author: Julien Resseguier, julien.resseguier@gmail.com

Sci. Adv. **9**, eadj0101 (2023)
DOI: 10.1126/sciadv.adj0101

The PDF file includes:

Figs. S1 to S13
Legends for movies S1 to S8

Other Supplementary Material for this manuscript includes the following:

Movies S1 to S8

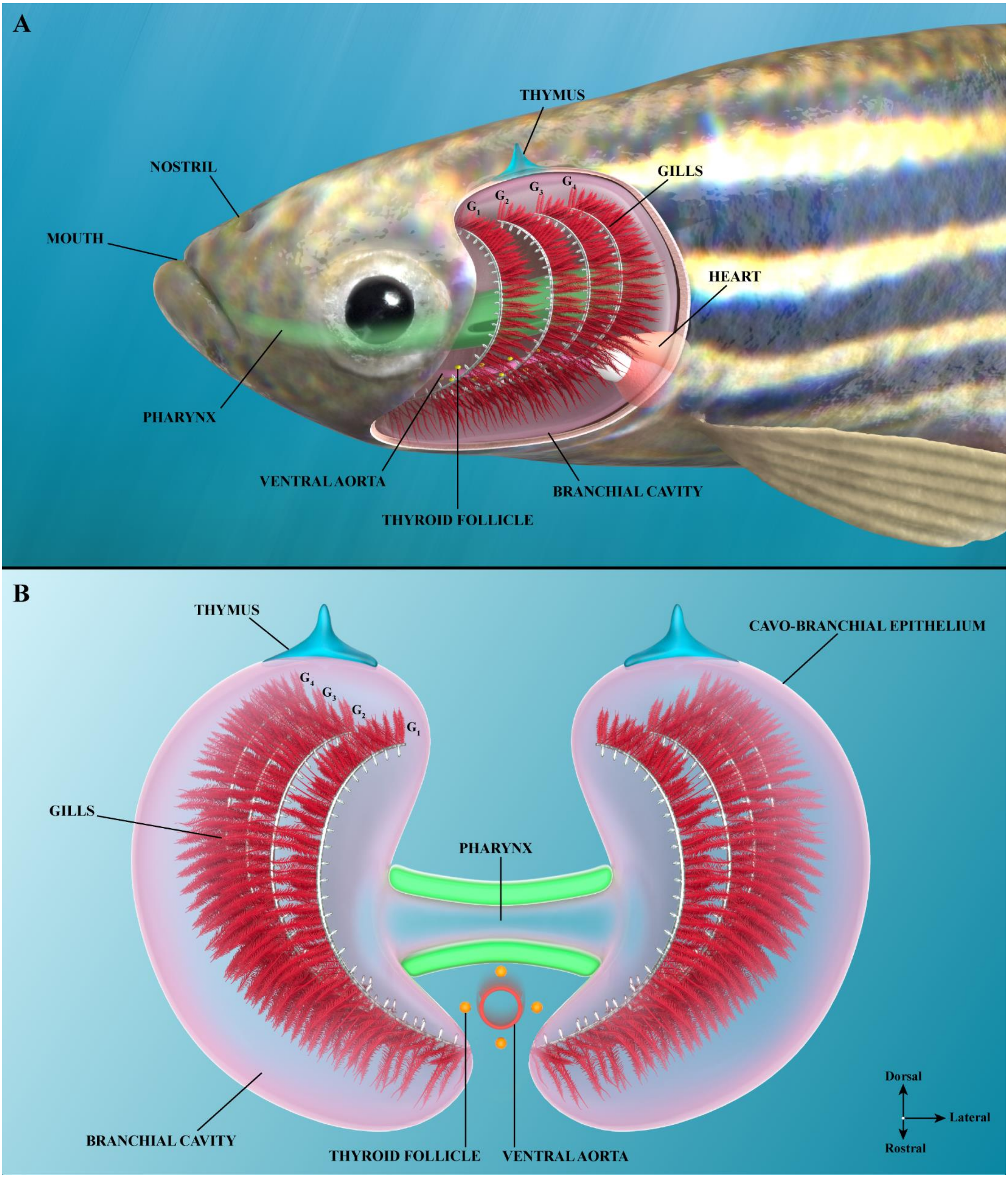

Figure S1 – ***Organization of the adult zebrafish branchial cavity***. Illustrations of the branchial cavity tissue organization as seen from the side (A) or from a front view (B). G_{1-4}: First to fourth gill arch. Illustrations made by Ella Maru studio.

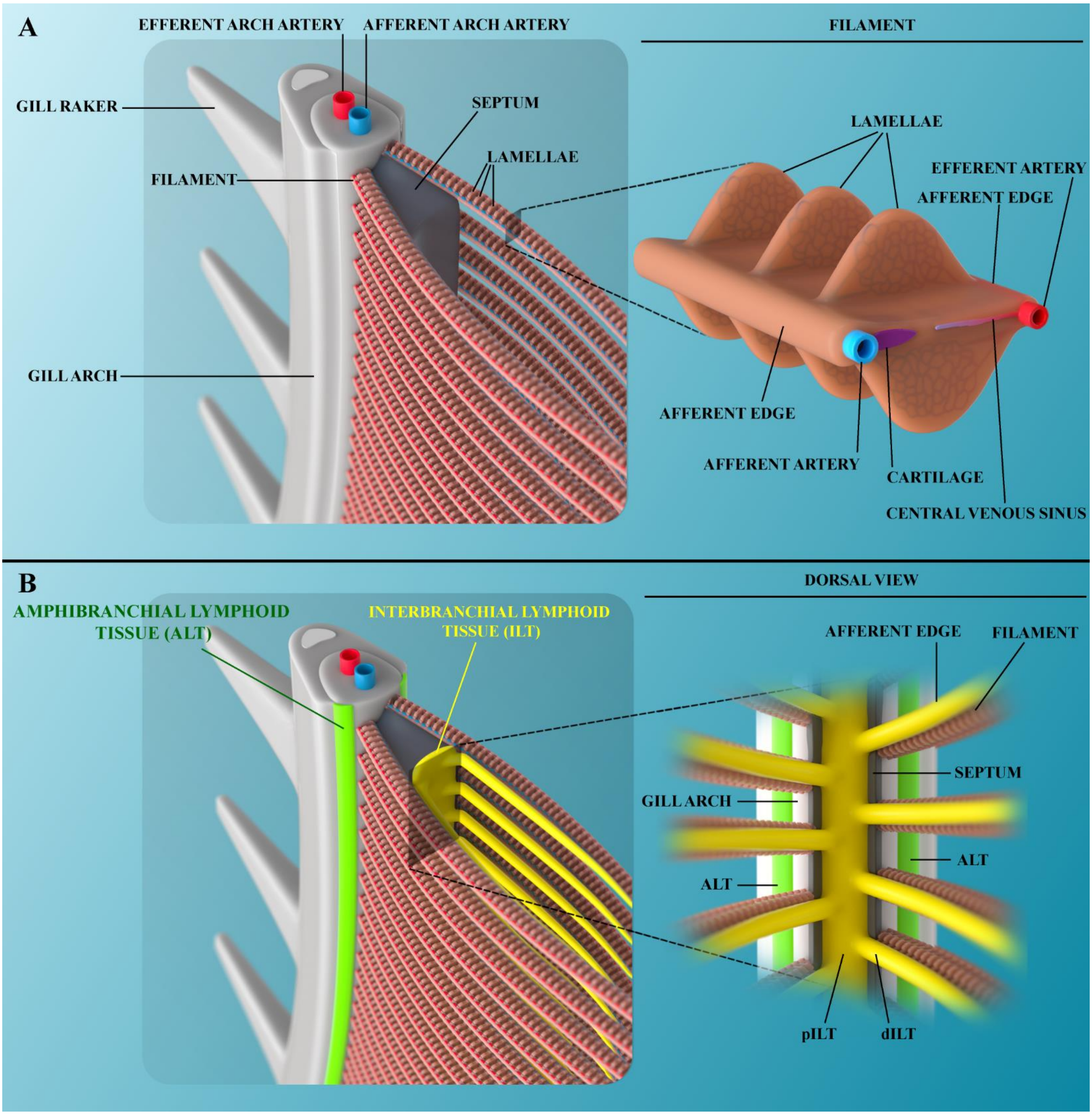

Figure S2 – ***Organization of the adult zebrafish gills***. Illustration of a gill arch (A) with its associated lymphoid tissues (B). Illustrations made by Ella Maru studio.

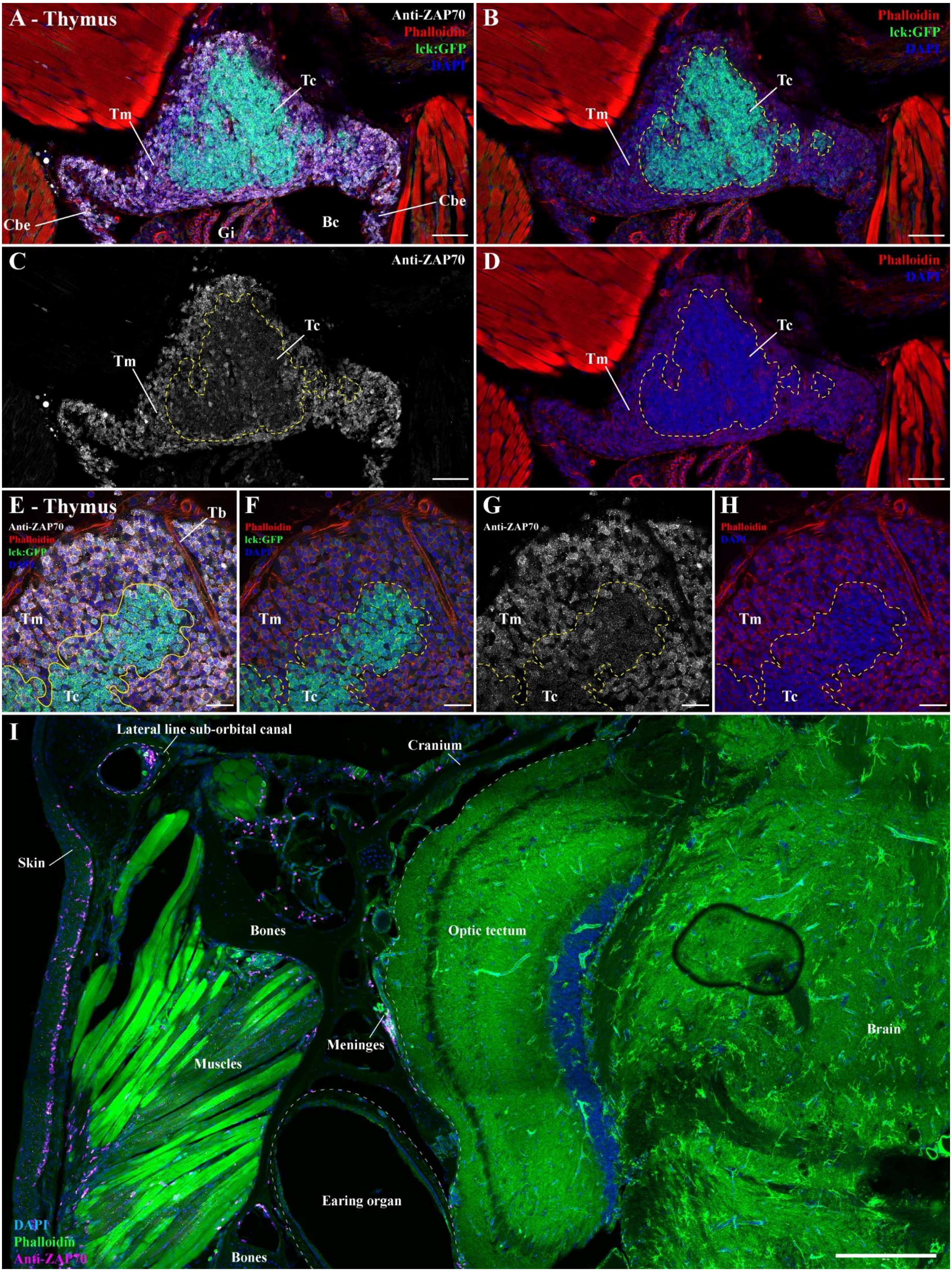
A - Thymus
Anti-ZAP70
Phalloidin
lck:GFP
DAPI
Tc
Tm
Cbe
Gi
Bc
Cbe
B
C
D
E - Thymus
Tb
F
G
H
I
Lateral line sub-orbital canal
Cranium
Skin
Bones
Optic tectum
Meninges
Muscles
Brain
Earing organ
Bones

Figure S3 – ***Specificity of the anti-ZAP70 labeling.*** (A-D) Cryosections from lck:EGFP adult zebrafish, in which T cells are fluorescent (green), labeled with anti-ZAP70 antibody (white). As expected, the thymus and its GFP-positive cells are labeled by the anti-ZAP70 labeling. In the thymus cortex thymocytes are intensely packed, highly express the gene lck and display a low anti-ZAP70 labeling. In contrast, the more developed thymocytes that populate the thymus medulla showed a lower lck gene expression and a higher anti-ZAP70 labeling. This distinction is even more striking at higher magnification (E-H). (I) In contrast to the thymus, cells labeled with the anti-ZAP70 antibody (magenta hot) are a lot less abundant in non-lymphoid tissues such as the brain. Annotations: Bc, Branchial cavity; Cbe, Cavobranchial epithelium; Gi, Gills; Tb, Trabecula; Tc, Thymus cortex and Tm, Thymus medulla. Scale bars: 200 µm (I), 50 µm (A-D) and 20 µm (E-H).

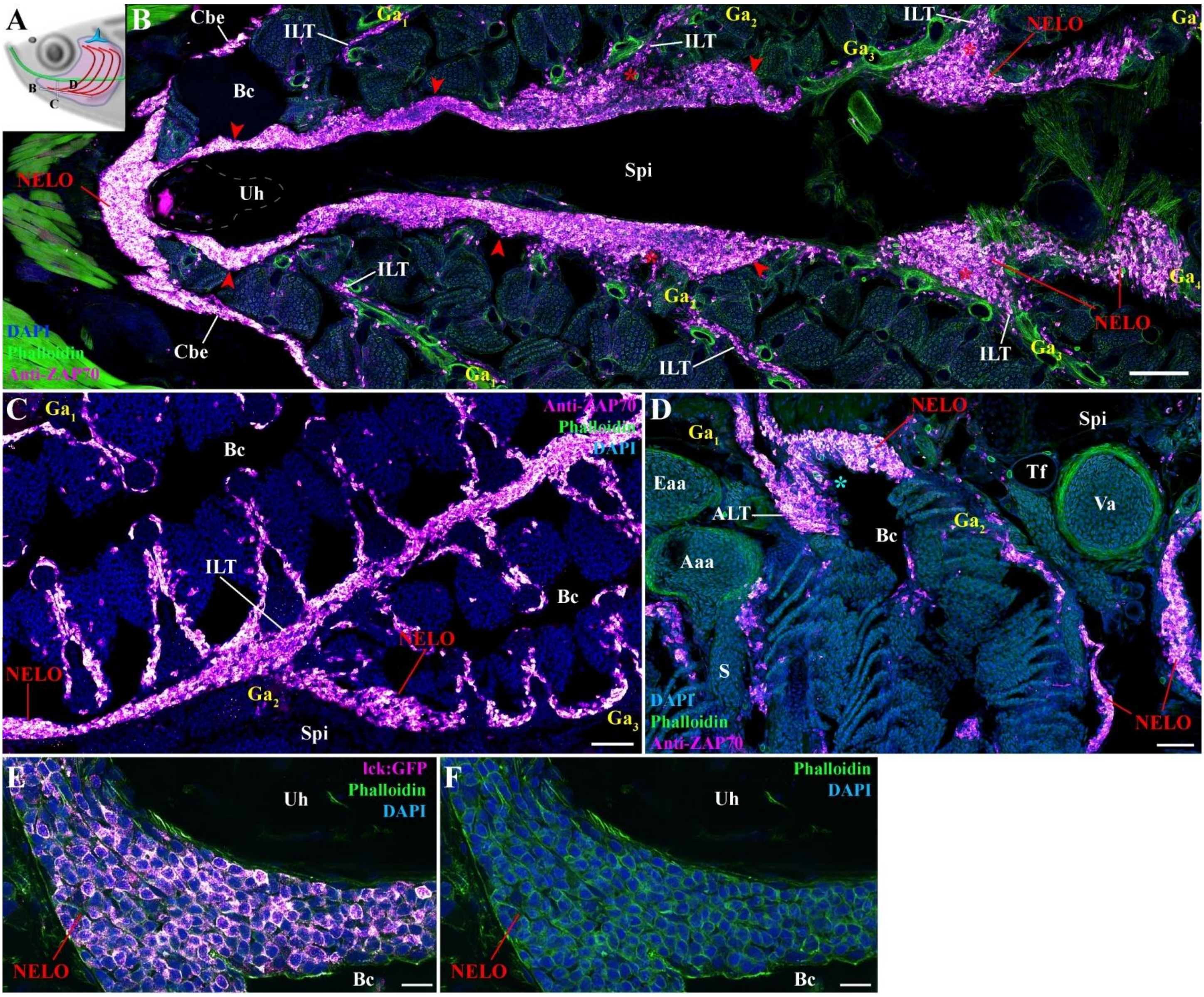

Figure S4 – ***Additional information on NELO's identification***. (A) Scheme localizing the section planes of images (B-D). (B) Additional 3D multi-field of view image of an adult zebrafish NELO from a branchial cavity coronal cryosection labeled with anti-ZAP70 (magenta hot). The structure corresponding to NELO is highlighted by red arrowheads. Connection sites between NELO and ILTs are marked by red stars. (C) Additional image illustrating the continuity between NELO and an interbranchial lymphoid tissue. (D) Additional image illustration the connection between NELO and an amphibranchial lymphoid tissue (cyan star). (E-F) NELO cryosection from a lck:EGFP adult zebrafish, in which T cells are fluorescent (magenta hot). Annotations: Aaa, Afferent arch artery; ALT, Amphibranchial lymphoid tissue; Bc, Branchial cavity; Cbe, Cavobranchial epithelium; Eaa, Efferent arch artery; Ga, Gill arch; ILT, Interbranchial lymphoid tissue; S, Septum; Spi; Sub-pharyngeal isthmus; Tf, Thyroid follicle; Uh, Urohyal bone and Va, Ventral aorta. Scale bars: 100 µm (B), 50 µm (C,D), and 10 µm (E,F).

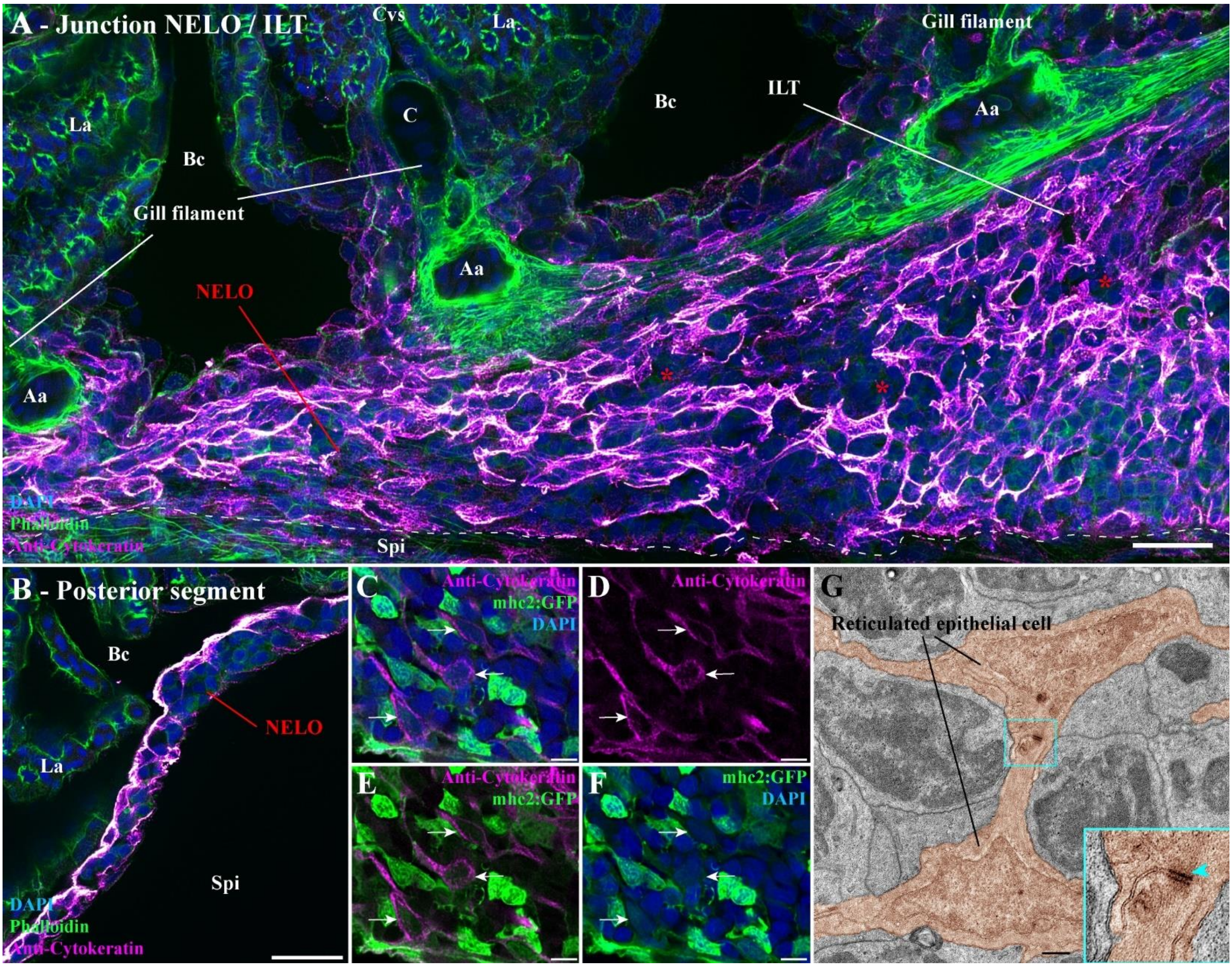

Figure S5 – ***Additional information on NELO's network of reticulated epithelial cells.*** (A) Adult zebrafish cryosections labeling with anti-cytokeratin (magenta hot) display the connection site of NELO with an ILT (red stars). (B) Network of reticulated epithelial cells at the posterior end of NELO. (C-F) Cryosection from a mhc2:GFP adult zebrafish, in which mhc2-expressing cells are fluorescent (green), labeled with anti-cytokeratin (magenta hot). NELO reticulated epithelial cells displayed a low mhc2 expression (white arrows). (G) Zoomed transmission electron micrograph from the ultrastructure map of Figure 2 highlighting the presence of an hemidesmosome (cyan arrowhead) at the junction of two reticulated epithelial cells (orange). Annotations: Aa, Afferent artery; Bc, Branchial cavity; C, Cartilage; ILT, Interbranchial lymphoid tissue; La, Lamellae; Spi, Sub-Pharyngeal isthmus. Scale bars: 20 µm (A,B), 5 µm (C-F), and 500 nm (G).

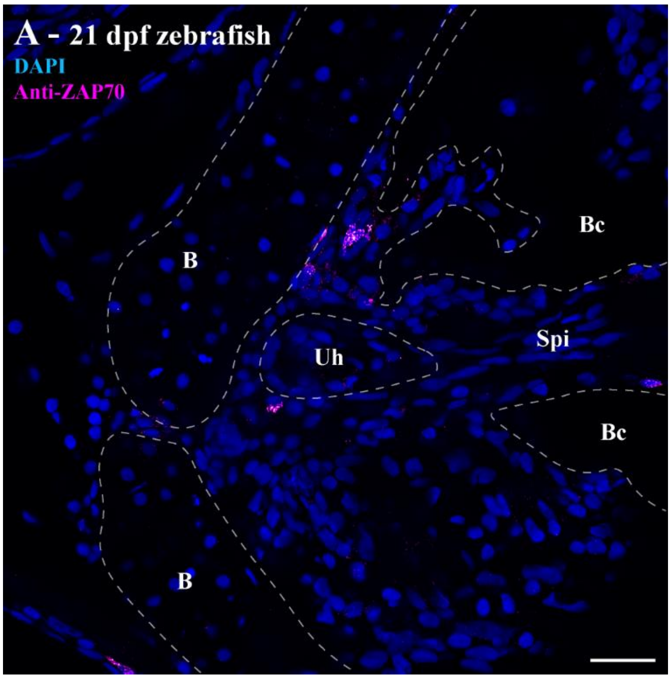

Figure S6 – ***Absence of NELO at 3 weeks post-fertilization.*** (A) Cryosection of the anterior sub-pharyngeal region of a 21 days post-fertilization zebrafish labeled with anti-ZAP70 (magenta hot) and DAPI (blue). At this age, the regions surrounding the sub-pharyngeal isthmus and the urohyal bones only display scarce amount of ZAP70 positive cells. Annotations: B, Bone; Bc, Branchial cavity; Spi, Sub-pharyngeal isthmus, and Uh, Urohyal bone. Scale bars: 20 µm (A).

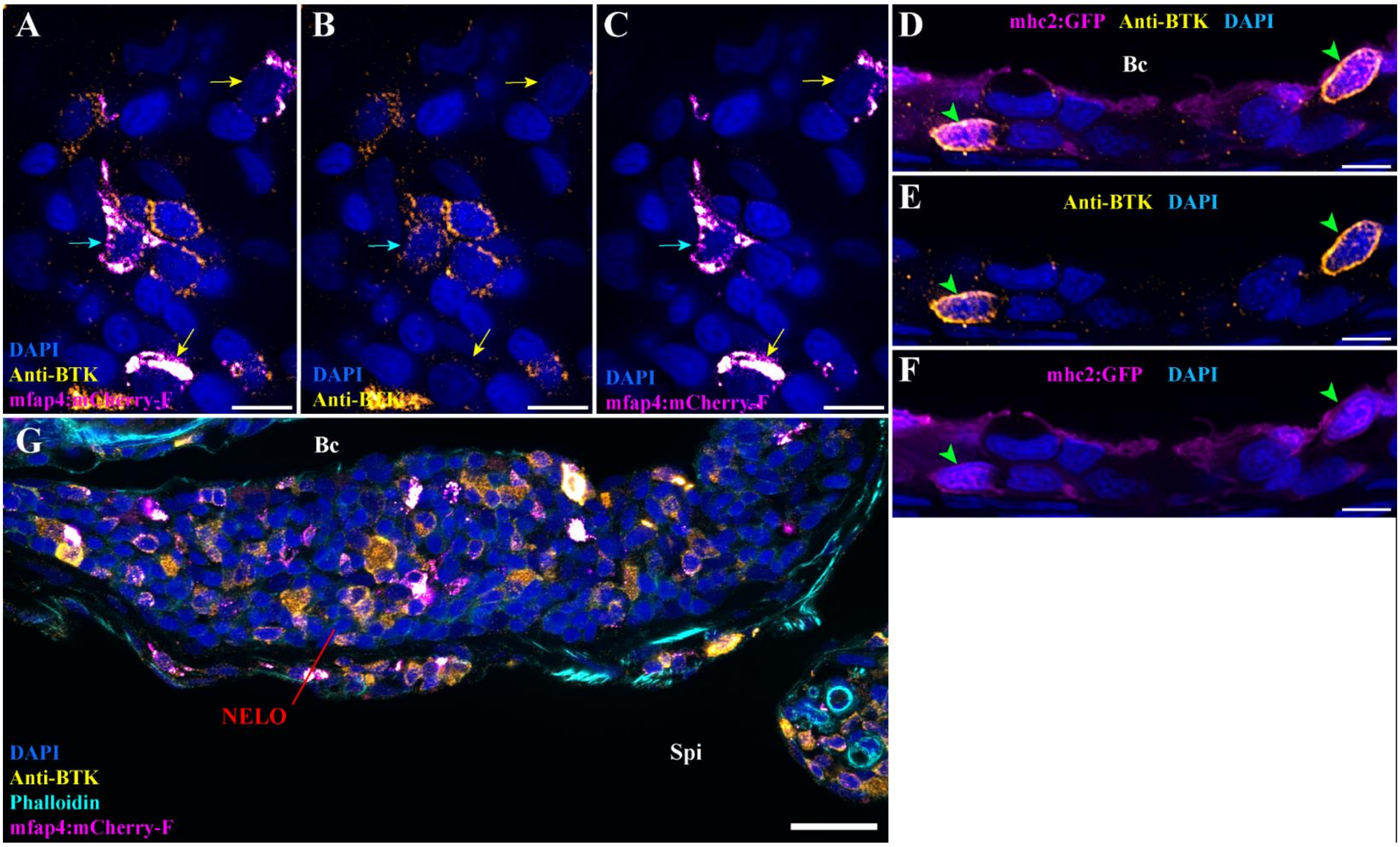

Figure S7 – ***Additional information anti-BTK antibody labeling***. (A-C) Anti-BTK labeling (orange hot) on mfap4:mCherry-F adult zebrafish cryosections, in which macrophage are fluorescent (magenta hot). Within NELO, both BTK-positive (cyan arrows) and BTK-negative (yellow arrows) macrophages are observed. (D-F) Cryosection from an mhc2:GFP adult zebrafish NELO, in which IgM expressing B cells are fluorescent (magenta hot), labeled with anti-BTK (orange hot). As expected, cells expressing IgM are also BTK-positive (green arrowheads). (G) Additional image displaying anti-BTK labeling in NELO of a mfap4:mCherry-F adult zebrafish. Annotations: Bc, Branchial cavity and Spi, Sub-pharyngeal isthmus. Scale bars: 20 µm (G), and 5 µm (A-F).

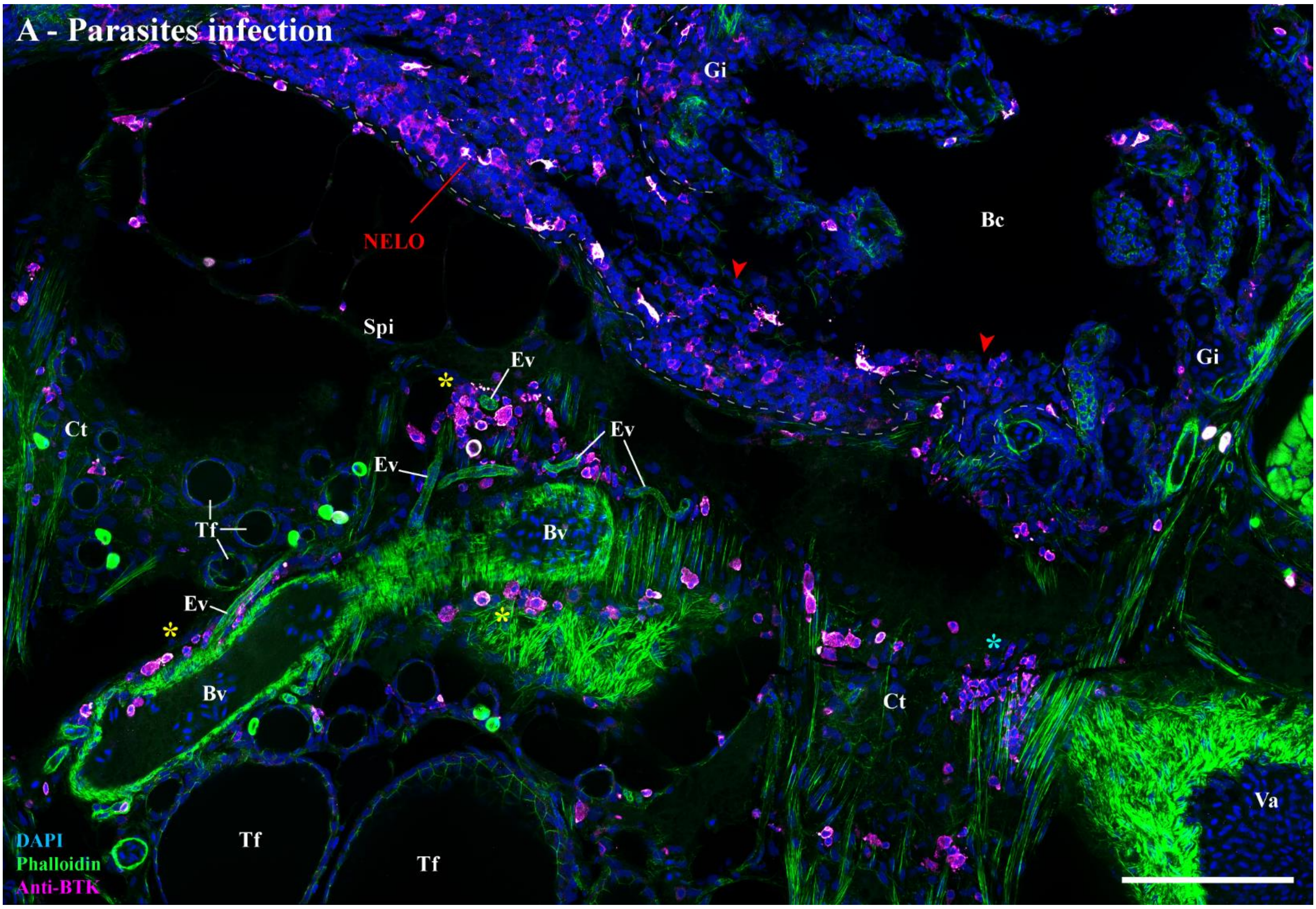

Figure S8 – ***Putative plasma/B cells clusters in parasites-infected adult zebrafish.*** (A) Cryosection from an adult zebrafish naturally co-infected with *Pseudoloma neurophilia*, *Pseudocapillaria tomentosa*, and *Myxidium streisingeri*, stained with phalloidin (green) and DAPI (blue), and labeled with anti-BTK antibody (magenta hot). In addition to putative BTK-positive plasma/B cells in NELO (red arrowheads), clusters of labeled cells were observed within the connective tissue (cyan star) and associated to endothelial vessels (yellow stars) of the sub-pharyngeal isthmus. Annotations: Bc, Branchial cavity; Bv, Blood vessel; Ct, Connective tissue; Ev, Endothelial vessel; Gi, Gills; Spi, Sub-pharyngeal isthmus; Tf, Thyroid follicle and Va, Ventral aorta. Scale bar: 100 µm.

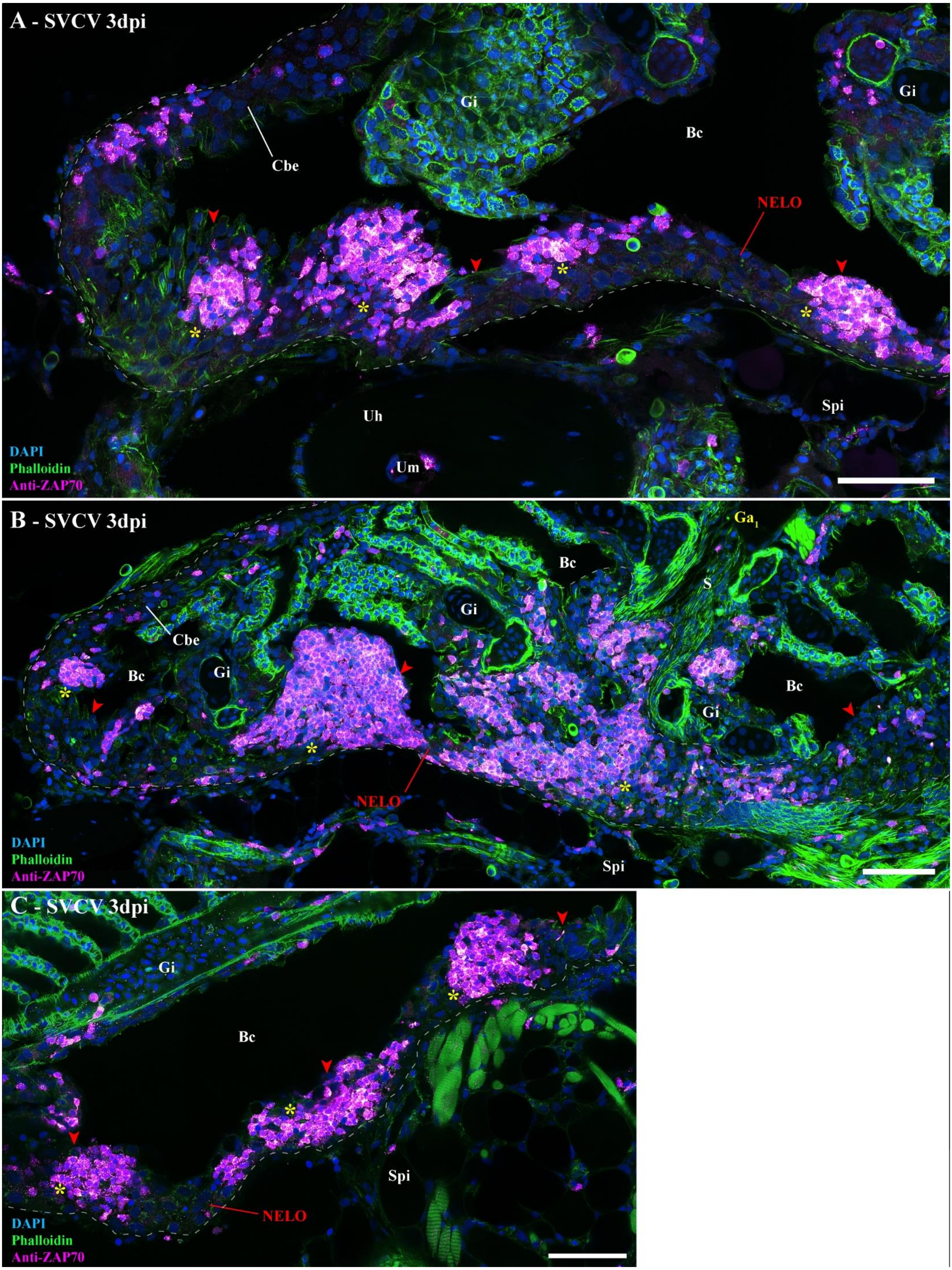
A - SVCV 3dpi
Gi
Bc
Cbe
Gi
NELO
Uh
Spi
Um
DAPI
Phalloidin
Anti-ZAP70
B - SVCV 3dpi
Ga1
Bc
S
Gi
Cbe
Bc
Gi
Bc
Gi
NELO
Spi
DAPI
Phalloidin
Anti-ZAP70
C - SVCV 3dpi
Gi
Bc
Spi
NELO
DAPI
Phalloidin
Anti-ZAP70

Figure S9 – ***Additional images on 3 days post-SVCV infection.*** (A-C) Cryosections from adult zebrafish three days after a 24h bath-infection with SVCV, stained with phalloidin (green) and DAPI (blue), and labeled with anti-ZAP70 (magenta hot). NELO (red arrowheads) displayed striking aggregation of T/NK cells into distinct clusters (yellow stars). Annotations: Bc, Branchial cavity; Cbe, Cavobranchial epithelium; dpi, day post-infection; Gi, Gills; Spi, Sub-pharyngeal epithelium; SVCV, Spring viremia of carp virus; Uh, Urohyal bone and Um, Urohyal marrow. Scale bars: 50 µm (A-C).

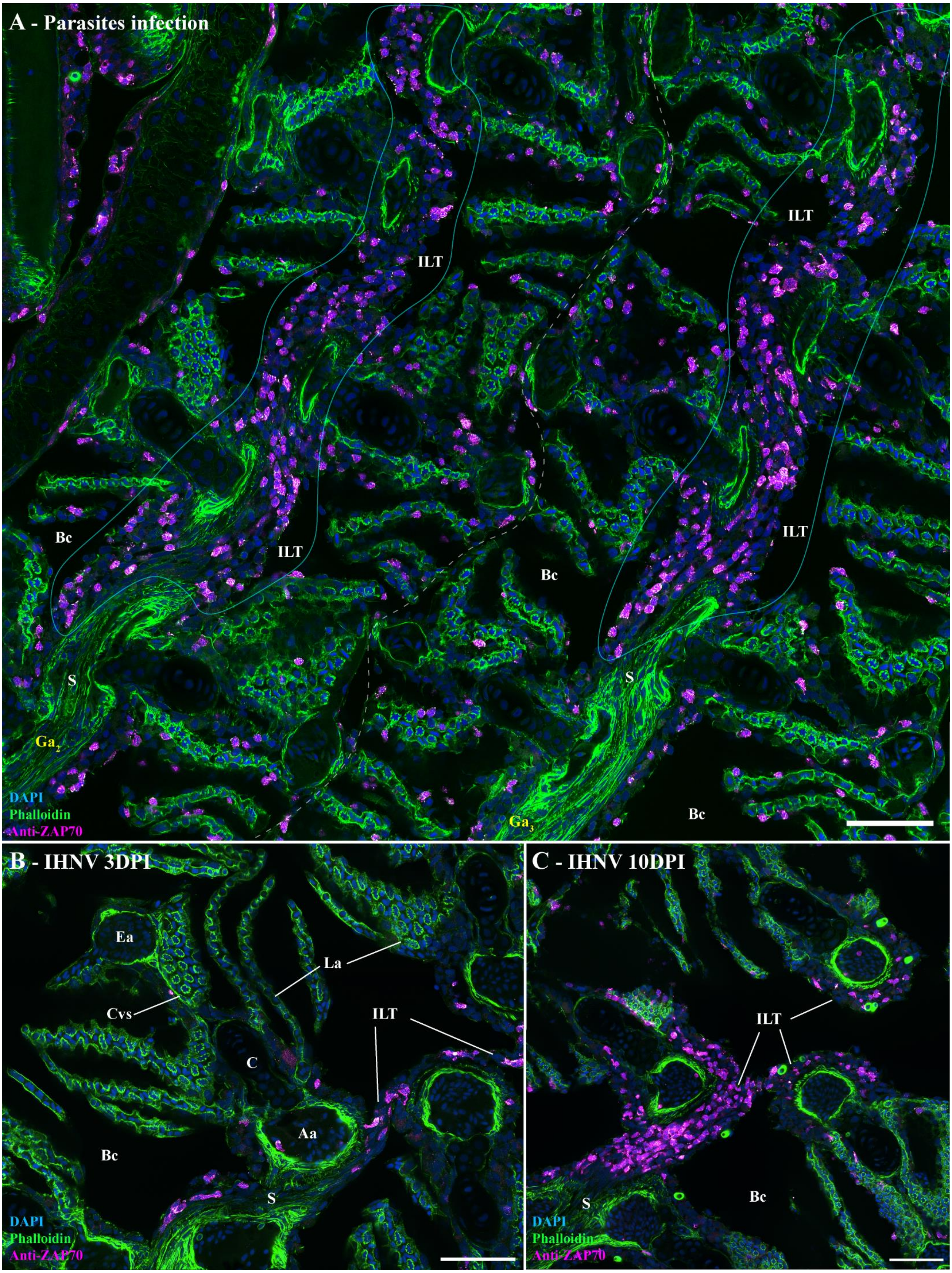
A - Parasites infection
ILT
ILT
ILT
ILT
Bc
Bc
Bc
S
S
Ga2
Ga3
DAPI
Phalloidin
Anti-ZAP70
B - IHNV 3DPI
Ea
La
Cvs
ILT
C
Aa
Bc
S
DAPI
Phalloidin
Anti-ZAP70
C - IHNV 10DPI
ILT
S
Bc
DAPI
Phalloidin
Anti-ZAP70

Figure S10 – ***Structural response of ILT to viral and parasitic infections.*** (A) Cryosections displaying the interbranchial lymphoid tissue of an adult zebrafish naturally co-infected with three parasitic diseases (*Pseudoloma neurophilia*, *Pseudocapillaria tomentosa*, and *Myxidium streisingeri*) stained with phalloidin (green) and DAPI (blue), and labeled with anti-ZAP70 antibody (magenta hot). The distribution of ZAP70-positive cells is more scattered than in uninfected fish and displayed small clusters of labeled cells. (B,C) Cryosections displaying the ILT of adult zebrafish 3 days (B) and 10 days (C) following a 24h bath-infection with IHNV. Although ILTs are severely depleted at 3 dpi, they appeared replenished at 10 dpi. Annotations: Aa, Afferent artery; Bc, Branchial cavity; C, Cartilage; Cvs, Central venous sinus; Ea, Efferent artery; Ga, Gill arch; ILT, Interbranchial lymphoid tissue; La, Lamellae and S, Septum. Scale bars: 50 µm (A-C).

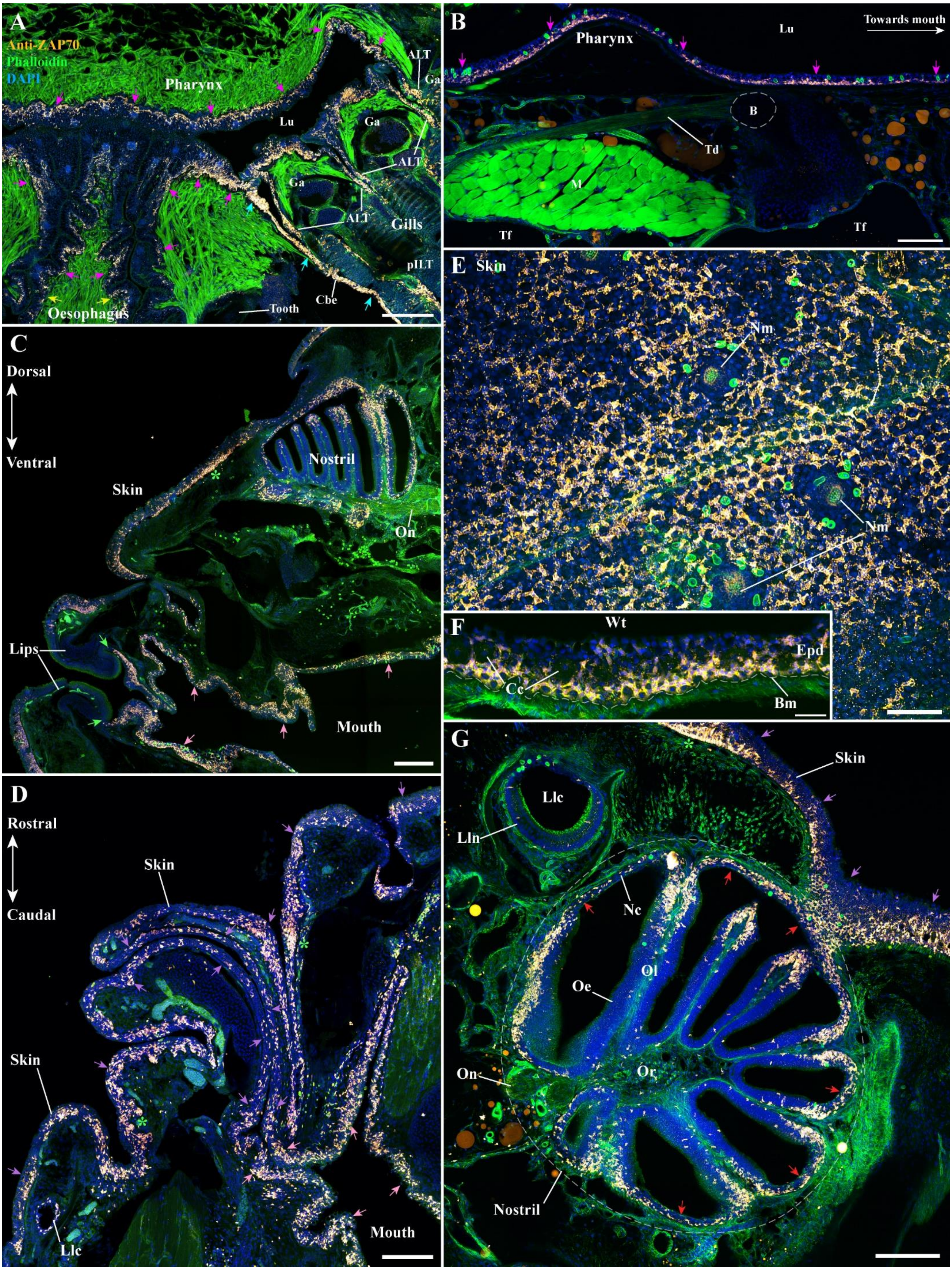

A
Anti-ZAP70
Phalloidin
DAPI
Pharynx
Lu
ALT
Ga
Gills
pILT
Cbe
Tooth
Oesophagus
B
Towards mouth
Pharynx
Lu
B
Td
M
Tf
C
Dorsal
Ventral
Skin
Nostril
On
Lips
Mouth
D
Rostral
Caudal
Skin
Llc
Mouth
E
Skin
Nm
F
Wt
Epd
Cc
Bm
G
Skin
Llc
Lln
Nc
Ol
Oe
On
Or
Nostril

Figure S11 – ***Extension of NELO's lymphoid network beyond the branchial cavity.*** (A-F) Cryosections from adult zebrafish stained with phalloidin (green) and DAPI (blue), and labeled with anti-ZAP70 (orange hot). (A) The lymphoid network of the branchial cavity, and which include NELO, is connected to the pharynx (magenta arrows) and oesophagus (yellow arrows) via T/NK cell-rich segments of the cavobranchial epithelium (cyan arrows). This lymphoid network is observed along the length of the pharynx (B – magenta arrows) and the mouth (C,D – pink arrows). Where it is absent from the keratinized lips of the fish (C – green arrows), it connected to the skin-associated lymphoid tissue (SALT) by the sides of the mouth opening (D - purple arrows). (E) Wholemount skin of a zebrafish head labeled with anti-ZAP70 and observed from above. The SALT is composed of a vast network of T/NK cells that are located at the basal layer of the epidermis and between club cells (F), and localized clusters of ZAP70-positive cells (C,D,G – green stars). (G) Via the organization of the SALT of the scale-less skin of the head, the lymphoid network observed in the branchial cavity is also continuous with the nasal-associated lymphoid tissue (NALT) (G – red arrows). Annotations: ALT, Amphibranchial lymphoid tissue; B, Bone; Bm, Basement membrane; Cbe, Cavobranchial epithelium; Cc, Club cells; Epd, Epidermis; Ga, Gill arch; Llc, Lateral line canal; Lln, Lateral line neuromast; Lu, Lumen; pILT, proximal Interbranchial lymphoid tissue; M, Muscles; Nc, Nasal cavity; Nm, Neuromast; Oe, Olfactory epithelium; Ol, Olfactory lamella; On, Olfactory nerve; Td, tendon; Tf, Thyroid follicle and Wt, Water. Scale bars: 200 µm (A,C), 150 µm (D,F), 100 µm (B), 50 µm (E), and 30 µm (E').

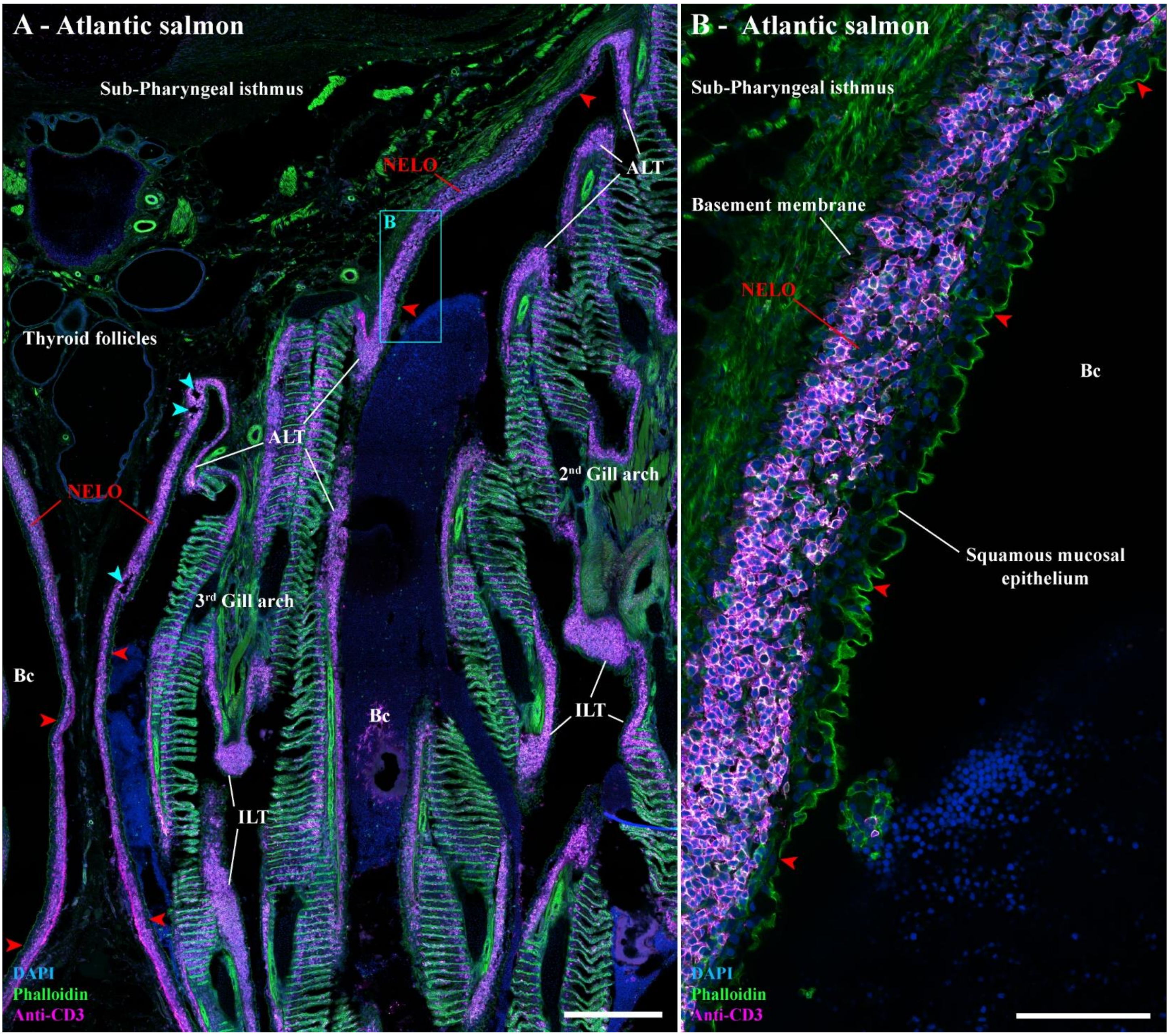

Figure S12 – ***Anti-CD3 labeling in Atlantic salmon NELO.*** (A) Transversal cryosection across the branchial cavity of an adult Atlantic salmon that is labeled with an anti-CD3 monoclonal antibody (magenta hot) and stained with phalloidin (green) and DAPI (blue). This immunolabeling shows that NELO (red arrowheads), the ILTs, and the ALTs are predominantly composed of CD3 positive T cells. Cyan arrowheads highlight the presence of aggregates of melanized cells. (B) Zoom from (A – cyan rectangle) displaying a single cell layer from NELO (red arrowheads). Annotations: ALT, Amphibranchial Lymphoid Tissue; Bc, Branchial cavity and ILT, Interbranchial Lymphoid Tissue. Scale bars: 500 µm (A) and 100 µm (B).

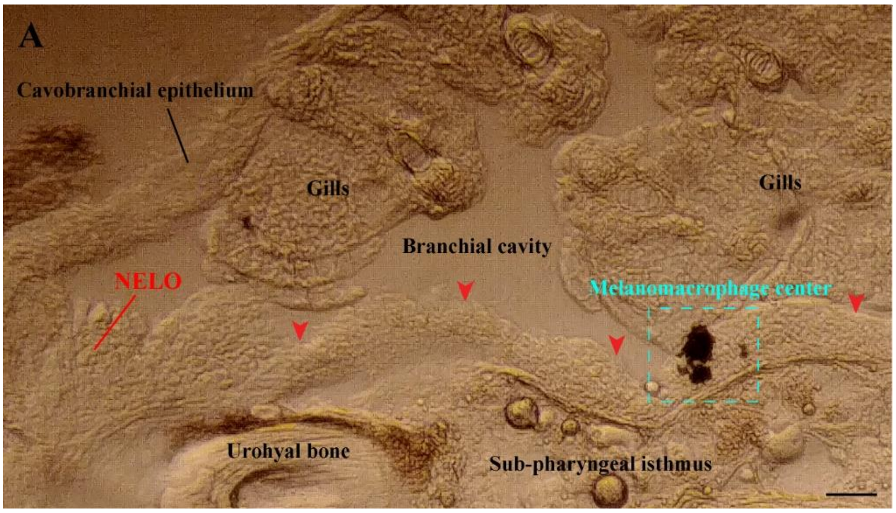

Figure S13 – ***Melanomacrophage center in zebrafish NELO.*** (A) Brightfield image of a cryosections displaying an aggregation of melanized cells (cyan rectangle) within NELO (red arrowheads). Scale bars: 50 µm (A).

Video S1 – ***3D reconstruction: zebrafish NELO.*** Reconstruction of NELO 3D structure using serial confocal tomography on a 15 wpf zebrafish head.

Video S2 – ***3D reconstruction: zebrafish branchial cavity region.*** Video displaying NELO (magenta), the ventral end of gill arches (green), the ALTs (cyan), and the thymus lobes (blue) that have been 3D reconstructed using serial confocal tomography on 15 wpf zebrafish head.

Video S3 – ***3D reconstruction: Sub-pharyngeal region of a zebrafish branchial cavity.*** Video displaying NELO (magenta), the ventral end of gill arches (green), the ALTs (cyan), and the thymus lobes (blue) that have been 3D reconstructed using serial confocal tomography on 15 wpf zebrafish head. A section plane has been included to highlight the sub-pharyngeal region located at the convergence of the gill arches.

Video S4 – ***3D reconstruction: Localization of NELO, ALTs, and thymus within a zebrafish head.*** Video displaying NELO (magenta), the ALTs (cyan), and the thymus lobes (blue) within the head of a 15 wpf zebrafish labeled with phalloidin (green).

Video S5 – ***3D reconstruction: NELO reticulated epithelial cell network.*** Reconstruction of a NELO reticulated epithelial cells network from a cryosection labeled with anti-cytokeratin (red) and DAPI (blue).

Video S6 – ***3D image: Endothelial vessels around zebrafish NELO.*** 3D image from a fli:GFP zebrafish cryosections, in which endothelial cells are fluorescent (green), stained with phalloidin (red) and DAPI (blue), and labeled with anti-ZAP70 (white). The video display an anterior region of NELO wrapped by endothelial vessels.

Video S7 – ***3D reconstruction: shared reticulated epithelial cell network between NELO and ILT.*** Reconstruction of a NELO reticulated epithelial cells network from the cryosection labeled with anti-cytokeratin (red) and DAPI (blue) presented **Fig.S3 A**.

Video S8 – ***3D image: Network of T/NK cells within the scale-less skin of a zebrafish head.*** The video displays the optical sections of a 3D image of the skin covering an adult zebrafish head. The acquisition was obtained from a wholemount head of zebrafish stained with phalloidin (green), DAPI (blue), and labeled with anti-ZAP70 (red hot). The optical sections are seen going from the exterior to the interior of the fish.
